# Phosphorylation of β-catenin at Serine552 correlates with invasion and recurrence of non-functioning pituitary neuroendocrine tumours

**DOI:** 10.1186/s40478-022-01441-5

**Published:** 2022-09-16

**Authors:** Ashutosh Rai, Soujanya D. Yelamanchi, Bishan D. Radotra, Sunil K. Gupta, Kanchan K. Mukherjee, Manjul Tripathi, Rajesh Chhabra, Chirag K. Ahuja, Narendra Kumar, Akhilesh Pandey, Márta Korbonits, Pinaki Dutta, Carles Gaston-Massuet

**Affiliations:** 1 Centre for Endocrinology, William Harvey Research Institute, Queen Mary University of London, London, UK; 2 Department of Endocrinology, Postgraduate Institute of Medical Education and Research, Chandigarh, India; 3 Molecular Biophysics Unit, Indian Institute of Science, Bangalore, India; 4 Departments of Histopathology, Postgraduate Institute of Medical Education and Research, Chandigarh, India; 5 Neurosurgery, Postgraduate Institute of Medical Education and Research, Chandigarh, India; 6 Radio-Diagnosis, Postgraduate Institute of Medical Education and Research, Chandigarh, India; 7 Radiotherapy, Postgraduate Institute of Medical Education and Research, Chandigarh, India; 8 Institute of Bioinformatics, Bangalore, India; 9 Institute of Genetic Medicine and Division of Proteomics, Mayo Clinic, Rochester, MN 55901 USA

**Keywords:** Phosphoproteomics, Non-functioning pituitary tumours, β-catenin-pSerine552, Prognostic

## Abstract

Non-functioning pituitary tumours (NF-PitNETs) are common intracranial benign neoplasms that can exhibit aggressive behaviour by invading neighbouring structures and, in some cases, have multiple recurrences. Despite resulting in severe co-morbidities, no predictive biomarkers of recurrence have been identified for NF-PitNETs. In this study we have used high-throughput mass spectrometry-based analysis to examine the phosphorylation pattern of different subsets of NF-PitNETs. Based on histopathological, radiological, surgical and clinical features, we have grouped NF-PitNETs into non-invasive, invasive, and recurrent disease groups. Tumour recurrence was determined based on regular clinical and radiological data of patients for a mean follow-up of 10 years (SD ± 5.4 years). Phosphoproteomic analyses identified a unique phosphopeptide enrichment pattern which correlates with disease recurrence. Candidate phosphorylated proteins were validated in a large cohort of NF-PitNET patients by western blot and immunohistochemistry. We identified a cluster of 22 phosphopeptides upregulated in recurrent NF-PitNETs compared to non-invasive and invasive subgroups. We reveal significant phosphorylation of the β-catenin at Ser552 in recurrent and invasive NF-PitNETs, compared to non-invasive/non-recurrent NF-PitNET subgroup. Moreover, β-catenin pSer552 correlates with the recurrence free survival among 200 patients with NF-PitNET. Together, our results suggest that the phosphorylation status of β-catenin at Ser552 could act as potential biomarker of tumour recurrence in NF-PitNETs.

**Supplementary Information:**

The online version contains supplementary material available at 10.1186/s40478-022-01441-5.

## Introduction

Pituitary tumours are the third most common primary intracranial tumour [1]. The prevalence of identifiable pituitary lesions varies from 14.4 (autopsy series) to 22.6% (radiological series) [2–6], while clinically relevant pituitary tumours are present in 0.1% of the general population. These tumours are benign, 35–40% grow invasively but rarely progress into true carcinomas [7]. Non-functioning pituitary tumours (NF-PitNETs) accounts for 30% of all clinically-relevant pituitary tumours [8]. NF-PitNETs do not result in clinical hypersecretion of hormones, but rather present with mass effects causing headache, visual impairment, and endocrine deficiencies [6]. Current treatment of symptomatic NF-PitNETs is either surgery with or without adjuvant radiotherapy [9, 10]. After complete tumour resection, the recurrence rate has been reported to be between 10 and 20%, while in cases with residual tumour after surgery, the recurrence rate is higher with a reported 40–50% recurrence within 5–10 years follow-up [11]. Tumour recurrence is a major factor of comorbidity in NF-PitNETs leading to poor clinical outcomes [12]. Indeed, approximately one third of patients with NF-PitNETs require multimodality treatment with repeat surgery or radiotherapy [13]. Radiotherapy has its own long-term consequences in the form of radiation-induced cerebrovascular damage, pan-hypopituitarism and second malignancies [14]. Moreover, tumour recurrence can be associated with panhypopituitarism, needing complete hormone replacement therapy [15]. Second surgery is also associated with increased surgical complications such as diabetes insipidus, cerebrospinal rhinorrhoea, meningitis, sinusitis and death as compared to primary surgery [10]. Therefore, being able to identify markers that predict recurrence at first surgery has important clinical diagnosis and prognostic value, particularly in a subgroup of patients that could be kept at close supervision. Biomarkers of tumour recurrence of NF-PitNETs that can predict the likelihood of recurrence are currently unknown. In order to identify possible biomarkers of NF-PitNETs’ recurrence, we undertook a quantitative phosphoproteomic analysis using liquid chromatography-mass spectrometry/mass spectrometry (LC–MS/MS) to identify the phosphopeptide patterns for each subgroup. We identified a cluster of proteins differentially phosphorylated in the recurrent NF-PitNET subgroup. Specifically, we identified that β-catenin at position Ser552 is more phosphorylated in recurrent NF-PitNETs and that the phosphorylation status of this residue correlates with recurrence free survival in a large cohort of NF-PitNET patients. These data suggest that phosphorylation of β-catenin at Ser552 could be used as potential biomarker for NF-PitNETs recurrence.

## Materials and methods

### Patients

Tumour samples from 20 male patients were used for the phosphoproteomic discovery phase. These patients underwent transsphenoidal surgery for clinically non-functioning pituitary neuroendocrine tumours in the Department of Neurosurgery, PGIMER, Chandigarh, were selected for this study after obtaining. Clinical and histopathological features are shown in Additional File 1: Table S1. 20 patients without any hormone excess clinically or biochemically were used in the study. Immunohistochemistry using pituitary hormones (GH, PRL, ACTH, LH, FSH, and TSH) and pituitary cell lineage transcription factors (SF1, PIT1, TPIT) established: 11 gonadotroph tumours (SF1-lineage PitNETs), 2 corticotroph tumours (TPIT-lineage PitNETs), 3 immature PIT1-lineage PitNETs (all PIT1+ve) consisting of 2 GH+ ve, one PRL + ve and 4 null cell tumours following the 5th edition of the WHO Classification of Endocrine and Neuroendocrine Tumours- PitNETs [16]. Ki-67 was < 3% in all tumours and they were negative for p53 immunostaining. We used fresh frozen tissue for mass spectrometry and immunoblotting. NF-PitNET patients were divided into three subgroups including non-invasive/non-recurrent (NI/NR n = 5), invasive (I n = 10), and recurrent subgroups (R n = 5). The non-invasive/non-recurrent (NI/NR) NF-PitNET subgroup, did not exhibit recurrences and hence was used as baseline to calculate the fold change of invasive and recurrent subgroups. Invasion was decided on the basis of pre-operative MRI using Knosp & Steiner classification [17]. We also considered histopathological invasion (invasion of mucosa, bone, and dura) and the surgeon’s finding (an intact medial wall of cavernous sinus (CS) as non-invasive while any disruptions of the medial wall of CS was considered as invasion by tumour) as one of the criteria [18, 19]. Non-invasive (NI) subgroup was defined as absence of invasion as per histopathological, radiological, and surgeon’s finding of intact medial wall of CS. Patients who were invasive for any of the criteria, radiological, surgeon’s finding, and histopathological were considered as invasive (I). All patients were followed-up with periodic MRI and recurrence was defined as an increase in tumour volume of  ≥ 20% or growth of ≥ 2 mm in any dimension and classified as recurrent.

Validation was performed by immunohistochemistry on tissue microarray derived from NF-PitNETs (n = 200) and somatotropinomas (n = 50; 8 sparsely granulated and 42 densely granulated) in quadruplets from patient samples operated in Postgraduate Institute of Medical Education and Research (PGIMER) between 2000 and 2015. Somatotroph tumours were included as controls to check the specificity of phosphoproteins identified in NF-PitNETs. Summary characteristics of these patients are shown in Additional File 1: Table S3. NF-PitNETs were classified according to the WHO Classification of Endocrine and Neuroendocrine Tumors–PitNETs [16] using immunohistochemistry against pituitary hormones (GH, PRL, ACTH, LH, FSH, and TSH) and pituitary transcription factors: SF1, PIT1, and TPIT which classified our NF-PitNETs tumour cohort in: 73.5% NF-PitNETs as gonadotroph tumour, 4.5% immature PIT1-lineage tumour (PIT1+ve), 7.5% corticotroph tumour (TPIT+ve), 8% as null cell tumours and 6.5% (plurihormonal tumour) (Additional File 2: Fig. S1a–d).

### Protein extraction and digestion

Tumour samples were subjected to lysis by sonication in 2% SDS buffer containing, phosphatase inhibitors such as 1 mM sodium fluoride, 2.5 mM sodium pyrophosphate and 1 mM sodium orthovanadate and 1 mM β-glycerophosphate. Subsequently, tissue lysates were obtained by centrifuging at 18,000 g at 4 °C for 20 min. Bicinchoninic acid (BCA) assay (Pierce, Illinois, USA, Cat #23,225) was performed to measure the protein amounts. Approximately 3 mg equivalent protein from each tissue was pooled in order to constitute a final protein amount of 15 mg in each NF-PitNET subgroup. The pooled lysates of four subgroups were subjected to reduction with 5 mM dithiothreitol (DTT) for 40 min at 60 °C and alkylation with 20 mM iodoacetamide (IAA) in dark for 15 min. Prior to proteolytic digestion, buffer exchange with 8 M Urea and 50 mM triethylammonium bicarbonate (TEABC) was carried out using 30 KDa filters (Millipore) and protein estimation was performed. Protein amounts were confirmed by normalization on SDS-PAGE across the four subgroups of NF-PitNET. Further, proteins were subjected to digestion with trypsin (Worthington Biochemical Corporation) for 16 h at 37 °C in 1:10 (w/w) ratio of enzyme to substrate. The efficiency of trypsin digestion was confirmed on a 10% resolving gel and continued with TMT labelling.

### TMT labelling and peptide fractionation

An equal amount of peptides from each condition were subjected to 4-plex tandem mass tags (TMT) labelling (Thermo Scientific) as per the instructions provided by the manufacturer [20]. Briefly, labelling was carried out as follows: non-invasive NF-PitNETs were labelled with 126 reporter ions, invasive NF-PitNETs with 127 and 129 reporter ions, and recurrent samples with 130 reporter ions. The reaction was incubated for 1 h at room temperature. Subsequently, labelling was quenched by incubating the labels with 5% hydroxylamine for 15 min. The labels were normalized, pooled and speed vacuum dried. Approximately 3 mg TMT labelled peptides were subjected to basic pH reverse-phase liquid chromatography (bRPLC) as described previously. The peptides were fractionated on an XBridge C_18_ column (5 μm, 250 × 4.6 mm) with a gradient of 2 to 35% of solvent B (7 mM TEABC in 90% acetonitrile (ACN). Around 50 μl of 1% formic acid was added to the 96 well plate prior to fraction collection to acidify the peptides. The fractions were collected on the 96-well plate and were pooled into a total of 12 fractions. The peptide fractions were lyophilized—until completely dried.

### Phosphopeptide enrichment

Each fraction was subjected to titanium dioxide (TiO_2_)-based phosphopeptide enrichment as described earlier [21]. Prior to phosphopeptide enrichment, the TiO_2_ beads (Titansphere, GL Sciences Inc) were activated by heating on dry bath at 95 °C for 15 min and the beads were resuspended in 2,5-dihydroxybenzoic acid (DHB) solution (80% ACN, 3% trifluoroacetic acid (TFA), and 5% DHB). Each peptide fraction was resuspended in 5% DHB solution and incubated with TiO_2_ beads at 2:1 ratio of peptide and beads for 30 min in a rotor at room temperature. Phosphopeptide-bound TiO_2_ beads were washed three times with wash solution containing 80% ACN and 3% TFA by centrifuging at 1500 g for 2 min. Phosphopeptide bound beads were then transferred to C_8_ column for elution. Peptides were eluted with 4% ammonia solution into tubes containing 40 μL of 4% TFA that were placed on ice. Finally, the peptides were dried and desalted using C_18_ Stage Tips. The eluted peptides were dried again and were subjected to LC–MS/MS analysis (Additional File 3: Fig. S2).

### LC–MS/MS analysis

LC–MS/MS analysis of enriched fractions of phosphopeptides was carried out in duplicates using Orbitrap Fusion Tribrid mass spectrometer and Orbitrap Velos, interfaced with Proxeon Easy-nLC 1000 system (ThermoFisher Scientific, Bremen, Germany). Each fraction was reconstituted in 0.1% formic acid and loaded on to a 2 cm long pre-column packed in-house with magic C18 AQ (Michrom Bioresources, Auburn, CA, USA). Peptides were then resolved on an analytical column (75 µ × 25 cm, 3 µ particle and 100 Å pore size) using a linear gradient of 5–30% of solvent B (0.1% formic acid in 95% acetonitrile) over 100 min. MS and MS/MS together was acquired using Orbitrap mass analyzer. Full scans were acquired with scan range of 400–1600 m/z and at a resolution of 120,000 at 400 m/z. Most intense precursor ions were selected at top speed data dependent mode and were fragmented using higher-energy collisional dissociation. Fragment ions were detected in Orbitrap with mass resolution of 30,000 and automatic gain control target value was set to 50,000 with maximum ion injection time of 200 ms. Singly charged ions were rejected and dynamic exclusion was set to 30 s. Internal calibration was carried out using lock mass option (m/z 445.1200025) from ambient air.

### Data analysis

LC–MS/MS data analysis was carried using Proteome Discoverer Platform, version 1.4.1.14 (ThermoFisher Scientific, Bremen, and Germany). The data was searched against NCBI Human RefSeq 70 database, which contained 35,298 unique protein sequences with known contaminants using SEQUEST and Mascot (Version 2.4) search algorithms. The search parameters used were set as indicated—precursor mass tolerance was set to 20 ppm and fragment mass tolerance to 0.05 Da. Carbamidomethylation of cysteine and TMT 6-plex (+ 229.163) modification at peptide N-terminus and lysine were set as fixed modification. Oxidation of methionine and phosphorylation at serine, threonine and tyrosine were set as variable modifications. Other search parameters include 1 missed cleavage by trypsin and 1% false discovery rate (FDR) at PSM level. PhosphoRS (Version 3.0) were used to calculate the confident localization of phosphosites for enriched phosphopeptides (Phospho RS score ≥ 75). Peptides with ratios ≥ 1.5 fold were considered as up-regulated and those with a ratio of ≤ 0.5 were considered as down-regulated for further bioinformatics analysis. Proteome Discoverer was used to calculate the fold changes by comparing the intensities of I and R subgroups with NI/NR. More than 50% increase (equals to 1.5 fold change) in intensity of a peptide in I and R in comparison to NI/NR.

### Bioinformatic analysis

The categorization of identified phosphorylated proteins in terms of molecular function, biological process and cellular component, and pathways were analysed using FunRich (Version 3.1.3) software [22].

### Data availability

The mass spectrometry data have been submitted to the ProteomeXchange Consortium (http://proteomecentral.proteomexchange.org) with data accession number PXD019269 [23].

### Immunohistochemistry (IHC)

IHC was carried out on tissue microarrays prepared from the 200 tumour samples form the validation cohort. Representative areas of NF-PitNETs were identified by a neuropathologist with extensive experience in pituitary histopathology. Each tumour area was biopsied with four 1 mm cores (4 each) and the cores were arranged in tissue microarray block. ICH was performed as previously described [24–26]; in short: paraffin-embedded tissue sections were deparaffinized and antigen retrieval carried out using citrate buffer (pH 6.0). Sections were incubated with primary antibodies [anti-phospho β-catenin Ser552 (CST# 9566) (1:300), anti-SF1 (Invitrogen #PA5-36,103) (1:200), anti-FSH-α (Bio-Rad #0100–0662) (1:300), anti-TPIT (Orb186399) (1:200), and anti-PIT1 (SC-442) (1:200) and anti-PRKAR1A Ser83 (Abcam #ab154851) (1:300). After washing, slides were incubated with horseradish peroxidase (HRP) conjugated secondary antibodies (BA-1000, Vector Laboratories, USA) and signal developed using 3,3’-diaminobenzidine (DAB) (SK-4100 Vector Laboratories, USA) and counterstaining was done with hematoxylin. Staining was scored as 0 (less than 5% of stained positive cells), 1+(5–30% of cells with positively stained), 2+ (31–60% of positive cells stained) and 3+ (greater than 60% of positive cell stained). The intensity and distribution scores were then summed for each case to calculate H-score [27]. Negative and positive tissue controls were used in each experiment.

### Immunofluorescence

Tumour samples from recurrent NF-PitNET patients (n = 3) were used for this part of the study. After transsphenoidal resection, tumor was washed with PBS (pH 7.4) and cells were dispersed using 2.5% Trypsin, Gibco, USA and mechanical dispersion procedure. Cell culture was performed in Dulbecco Modified Eagle Media (DMEM, Gibco, USA) containing fetal calf serum (FCS, Gibco, USA), penicillin and streptomycin at 37 °C and 5% CO2.For immunofluorescence cells, were incubated with primary antibodies [anti-phospho β-catenin Ser552 (# 9566) (1:300), and anti-CD44 (ab157107) (1:100)] as described in [28, 29]. After washing, cells were incubated with fluorochrome conjugated secondary antibodies. Cell nuclei were stained with 4′,6-diamidino-2-phenylindole (DAPI) and visualised under fluorescence microscope (Evos, Thermo Fisher Scientific, Waltham, MA USA).

### Immunoblots

A total of 30 μg equivalent amount of protein per sample were loaded on 10% SDS–PAGE gel and transferred to nitrocellulose membranes for further processing. The membrane was blocked with 5% bovine serum albumin (BSA) for 1 h at room temperature, followed by overnight incubation at 4 °C with the primary antibodies [phospho β-catenin Ser552 (CST #9566) (1:500), β-catenin (Abcam #ab32572) (1:500)]. Membranes were incubated with appropriate peroxidase-conjugate secondary antibodies (Santa Cruz, USA, 1:3000) and bands were visualized by the enhanced chemiluminescence (ECL) method (BioRad, USA).

### Statistical analysis

Data are presented as the mean ± standard deviation of the mean (SD), unless specified. Data were checked for normality using Kolmogorov–Smirnov test. Normally distributed data were compared using unpaired *t*-test, while skewed data were compared using Mann Whitney test, and Pearson’s correlation test. *P*-values < 0.05 were considered as statistically significant. Hierarchical clustering analysis was done using open-source Multi experiment Viewer 4 (MeV) software. Categorical data were compared using Fischer’s exact test. We also did multiple *t*-test and plotted volcano plot. In order to study the impact of the phospho β-catenin Ser552, H-scores (continuous variables) were categorized to allow Kaplan–Meier analysis. The scores were categorized as follow: patients with H-score above 160 (cut-off based on maximum sum of sensitivity and specificity) were encoded as “1”, whereas the remainder were encoded as “0”. Statistical were performed using Graph Pad Prism 9 (San Diego, USA).

## Results

### Phosphoproteome of NF-PitNETs

**Fig. 1:**
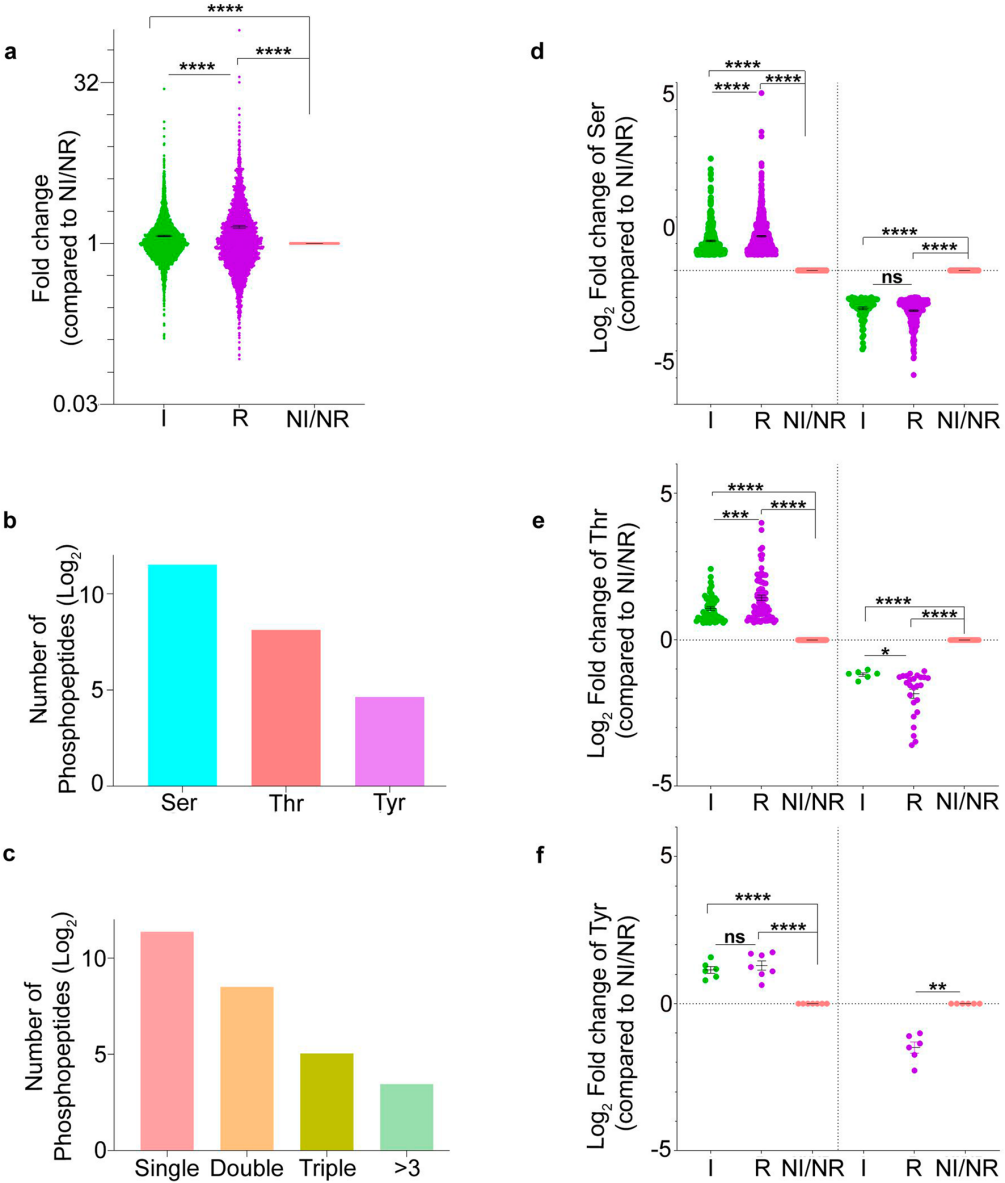
Overview of the phosphoproteome of NF-PitNETs. **a** Graphical quantification of the phosphopeptides fold change among NF-PitNETs subgroups: invasive (I, green), recurrent (R, purple) and non-invasive/non-recurrent (NI/NR, pink). Statistical analysis reveals differentially expressed phosphopeptides between I, R, and NI/NR subgroups. Note that fold change is compared to non-invasive/non-recurrent NF-PitNETs subgroup. **b** Bar graph indicating the overall proportion of phosphoserine, phosphothreonine and phosphotyrosine sites in the NF-PitNETs phosphoproteome. Overview of phosphorylation sites per amino acid shows that Ser is the most phosphorylated amino acid followed by threonine (Thr) and tyrosine (Tyr). **c** Bar graph representing the number of phosphopeptides carrying either a single phosphosite, double phosphosites, triple and more than three phosphosites. **d-e** The phosphorylation of Ser and Thr is significantly upregulated in recurrent compared to invasive and NI/NR **f** No differences were found in the phosphorylation status of Tyr residues among the I and R, NF-PitNETs subgroups. Abbreviations: I, invasive; R, recurrent; Ser, serine; Thr, threonine; Tyr, tyrosine. **d**–**f** Student’s *t*-test from experimental triplicates, *p < 0.05, **p < 0.01, ***p < 0.001, ****p < 0.0001

In our study, we present the full NF-PitNET phosphoproteome containing 3185 quantified phosphopeptides. We found significant differences in phosphorylation stoichiometry between invasive (I), recurrent (R) and non-invasive/non-recurrent (NI/NR) disease subgroups (*p* < 0.0001). The mean fold change for the R (1.43 ± 0.04) and I (1.17 ± 0.01) was significantly different than the NI/NR subgroup (Fig. 1a *p* < 0.0001). The frequency of identified phosphosites were: Ser (90.3%; log_2_ = 11.5), Thr (8.9%; log_2_ = 8.1), and Tyr (0.8%; log_2_ = 4.6) (Fig. 1b). From the 3185 identified peptides, 88% (log_2_ = 11.5) were phosphorylated at single residue, 10% (log_2_ = 8.3) at two residues, 0.43% (log_2_ = 3.8) at three residues, whilst 0.22% (log_2_ = 2.8) were found phosphorylated at multiple sites (> 3) (Fig. 1c). We identified that the proportion of phosphorylated peptides was different among I and R subgroups in comparison with NI/NR (Additional File 4: Fig. S3a–c). Specific phosphorylated residue analysis indicated that Ser and Thr sites were more phosphorylated in I and R compared to NI/NR subgroups of NF-PitNETs (Fig. 1d–f). The Pearson Correlation Coefficient test (r) has been widely used to verify the reproducibility of phoshoproteome data [30, 31]. Pearson’s correlation coefficient (r) of 0.78 and 0.73 for I and R replicates, showed reproducibility and robustness of our experimental data (Additional File 4: Fig. S3d & e). Moreover, our results showed that despite there is some overlap in the phosphorylation pattern between different NF-PitNETs subgroups, there is a substantial quantitative difference in the phosphoproteome that characterises each specific NF-PitNET subgroup. For our study, we focused on Class I phosphopeptides for further analyses as Class I phosphopeptide have higher degree of validity because each site has a localization probability for the phospho-group of at least 75% [32].

### Differential phosphoprotein signature characterises NF-PitNET subgroups

To distinguish between changes in phosphorylation across the NF-PitNET subgroups, a 1.5 fold change ratio between NI/NR and the other two groups was considered (Additional File 5: Fig. S4a, b). This analysis showed that in the invasive group, a total of 566 phosphorylation sites were altered, of which 83.7% were phosphorylated and 16.2% were hypo-phosphorylated. The recurrent group was biologically most active with 1113 altered phosphosites. In this group, 71.1% sites were phosphorylated and 28.8% were hypo-phosphorylated. Principal component analysis also showed variance in phosphorylation across the NF-PitNET subgroups (Additional File 5: Fig. S4c). In our study, only phosphopeptides identified in triplicates (n = 110) were selected for bioinformatic analysis and further validation (Additional File 1: Table S3).

### Hierarchical clustering identifies phosphorylated proteins in recurrent NF-PitNETs

**Fig. 2:**
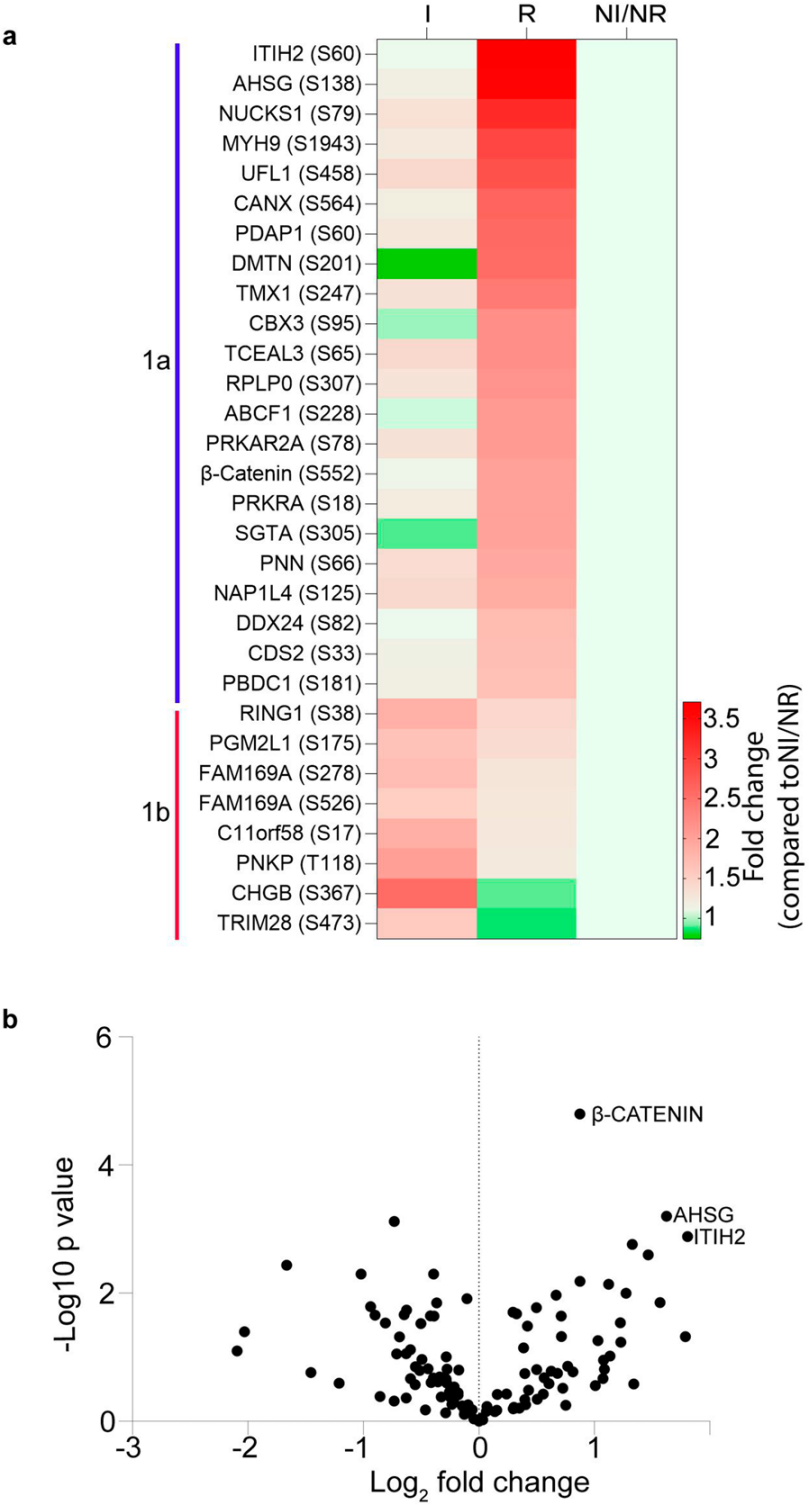
Heat map reveals differential clustering of phosphoproteins among NF-PitNET subgroups. **a** The fold change values of 30 differentially regulated phosphoproteins were grouped by an unsupervised hierarchical clustering using Pearson’s correlation distance and average linkage. Proteins with elevated phosphorylation levels are indicated by red while decrease in phosphorylation is shown by green. Each row represents a phosphoprotein (phosphosites shown in brackets) and each column a NF-PitNET subgroup: invasive (I), recurrent (R) and non-invasive/non-recurrent (NI/NR). **b** Volcano plot, obtained from quantitative mass spectrometry, indicating differentially phosphorylated protein in recurrent (R) NF-PitNETs. The Log_2_ fold change indicates the mean phosphorylation level of proteins. Each dot represents a phosphoprotein. Multiple-*t* test shows significant hyperphosphorylation of β-Catenin (*p* = 0.000016), AHSG (*p* = 0.0006), and ITIH2 (*p* = 0.001) in recurrent NF-PitNETs

We investigated the global heterogeneity of NF-PitNETs by unsupervised hierarchical clustering of all significantly quantified phosphopeptides. The hierarchical matrix has three columns representing I, R, and NI/NR disease subgroups. Row wise matrix was divided into two main groups 1a and 1b: one cluster comprising phosphoproteins phosphorylated in recurrent group (1a) and another cluster (1b) contains proteins hyperphosphorylated in invasive group (Fig. 2a) compared to NI/NR. This co-segregation was also represented by the principal component analyses of the NF-PitNETs phosphoproteome (Additional File 5: Fig. S4c). Volcano plot of the NF-PitNET phosphoproteins revealed β-catenin (*CTNNB1* gene; *p* = 0.000016), Inter-alpha-trypsin inhibitor heavy chain H2 (ITIH2; *p* = 0.001) and Alpha-2-HS-glycoprotein (AHSG; *p* = 0.0006) as significantly more phosphorylated proteins in the recurrent tumours compared to the invasive NF-PitNET subgroup (Fig. 2b).

**Fig. 3:**
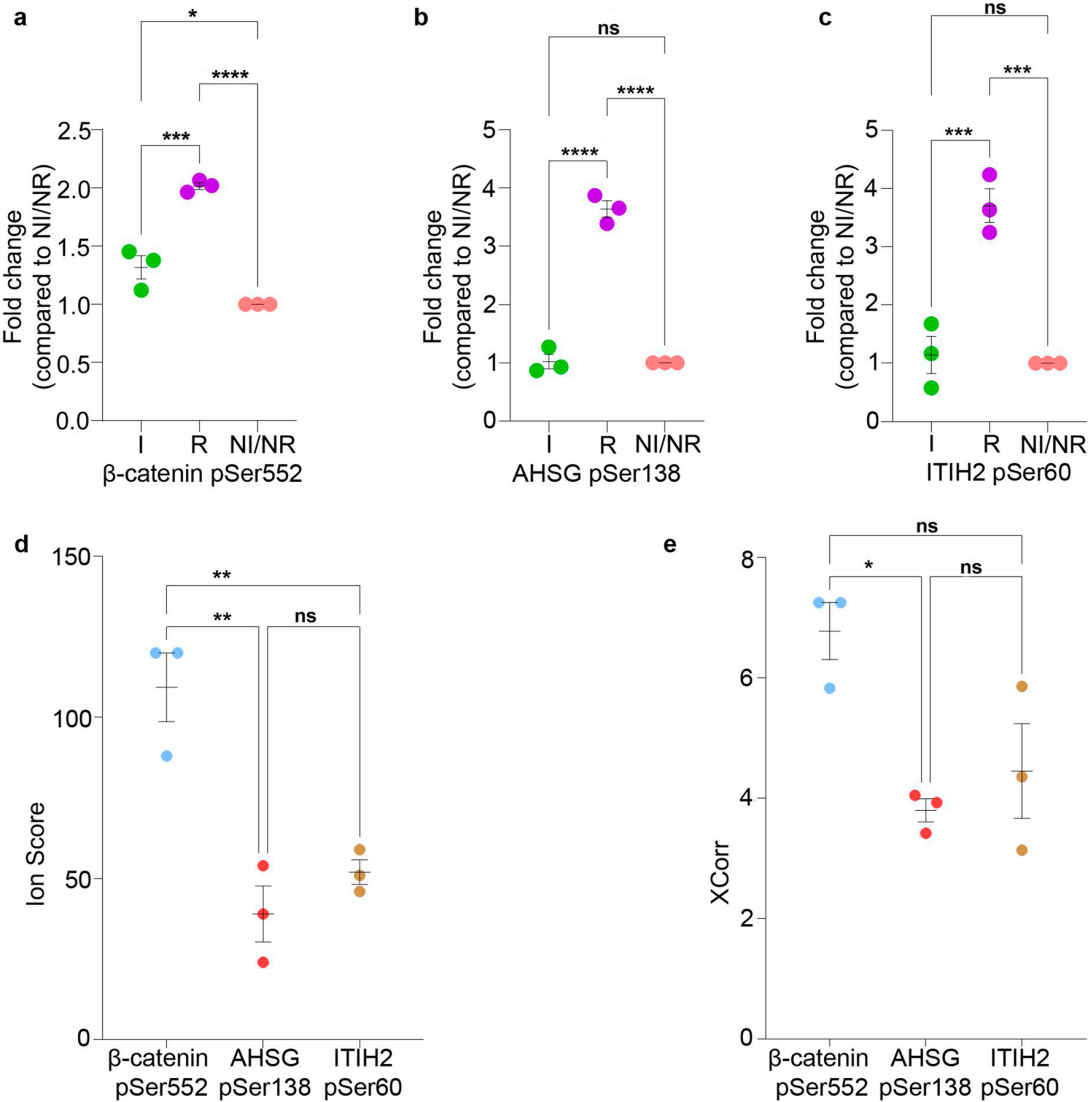
Elevated phosphorylation of β-catenin pSer552, AHSG pSer138, and ITIH2 pSer60 in recurrent NF-PitNETs. Phosphoprotein profiles in recurrent NF-PitNET showed significant increase in phosphorylation of β-catenin pSer552 (**a**), Alpha-2-HS-glycoprotein (AHSG) pSer138 (**b**), and Inter-alpha-trypsin inhibitor heavy chain H2 (ITIH2) pSer60 (**c**) in recurrent (R) NF-PitNETs subgroup compared to invasive (I) and non-invasive/non-recurrent (NI/NR). (**d**) Graphical representation of the Ion Score (a measure of how well the observed MS/MS spectrum matches the stated peptide) shows highest Ion Score for β-catenin pSer552 compared to AHSG pSer138 and ITIH2 pSer60. (**e**) Graphical representation of XCorr (cross correlation). Values above 2.0 indicate of a good fit of experimental peptide fragments to theoretical spectra. XCorr was significantly high for β-catenin pSer552 compared to other phophoproteins. Abbreviations: NI/NR, non-invasive/non-recurrent; I, invasive; R, recurrent. A two-tailed unpaired Student’s *t*-test was performed *p < 0.05, **p < 0.01, ***p < 0.001, ****p < 0.0001

Although our analyses identified 30 differentially phosphorylated peptides across the NF-PitNET subgroups (invasive and recurrent) (Fig. 2), phosphorylation of β-catenin pSer552, ITIH2 pSer60, and AHSG pSer138 were found increased exclusively in the recurrent group (Fig. 3a–c). Importantly, these phosphopeptides exhibited high Ion-Score and Xcorr, indicating that these peptides were identified confidently [33, 34]. Moreover, β-catenin pSer552 exhibited the highest Ion Score and Xcorr among all the identified peptides, making of β-catenin pSer552 a stronger candidate of NF-PitNET recurrence and validation (Fig. 3d–e).

In silico analyses using FunRich software for protein–protein interaction network identified β-catenin and Wnt signalling pathways components as differentially phosphorylated (Additional File 6: Fig. S5d & Additional File 7: Fig. S6). Several studies have reported the deregulation of the Wnt pathways in PiNETs [35, 36]. Due to the importance of β-catenin in tumour development we centred our study on the validation of β-catenin phosphorylation in recurrent NF-PitNETs.

### β-catenin pSer552 correlates with recurrence in NF-PitNETs

**Fig. 4:**
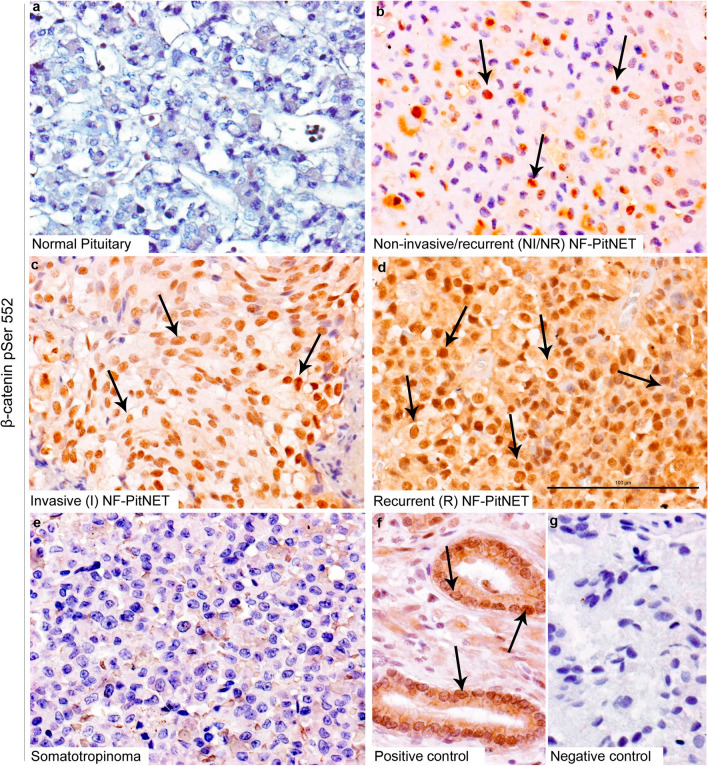
Increased phosphorylation of β-catenin at pSer552 in recurrent and invasive NF-PitNETs. **a-f** Immunohistochemistry against β-catenin pSer552 in normal pituitary (**a**), non-recurrent/non-invasive NF-PitNET (**b**), invasive (**c**), recurrent (**d**), and somatotropinoma (**e**). Note that β-catenin pSer552 showed strong nuclear positivity in recurrent and invasive adenomas (black arrows in **c** and **d**). No expression of β-catenin pSer552 was found in normal pituitary (**a**) and somatotropinomas (**e**). Prostate carcinoma was used as positive control (**f**). Positive staining of β-catenin pSer552 is shown by black arrows (**f**) while omission of primary antibody was used as negative control. Negative nuclei are shown as blue (counterstaining by hematoxylin (**g**). Positive staining shown by brown colour is marked by black arrows. Scale bar in d represents 100 µm

**Fig. 5:**
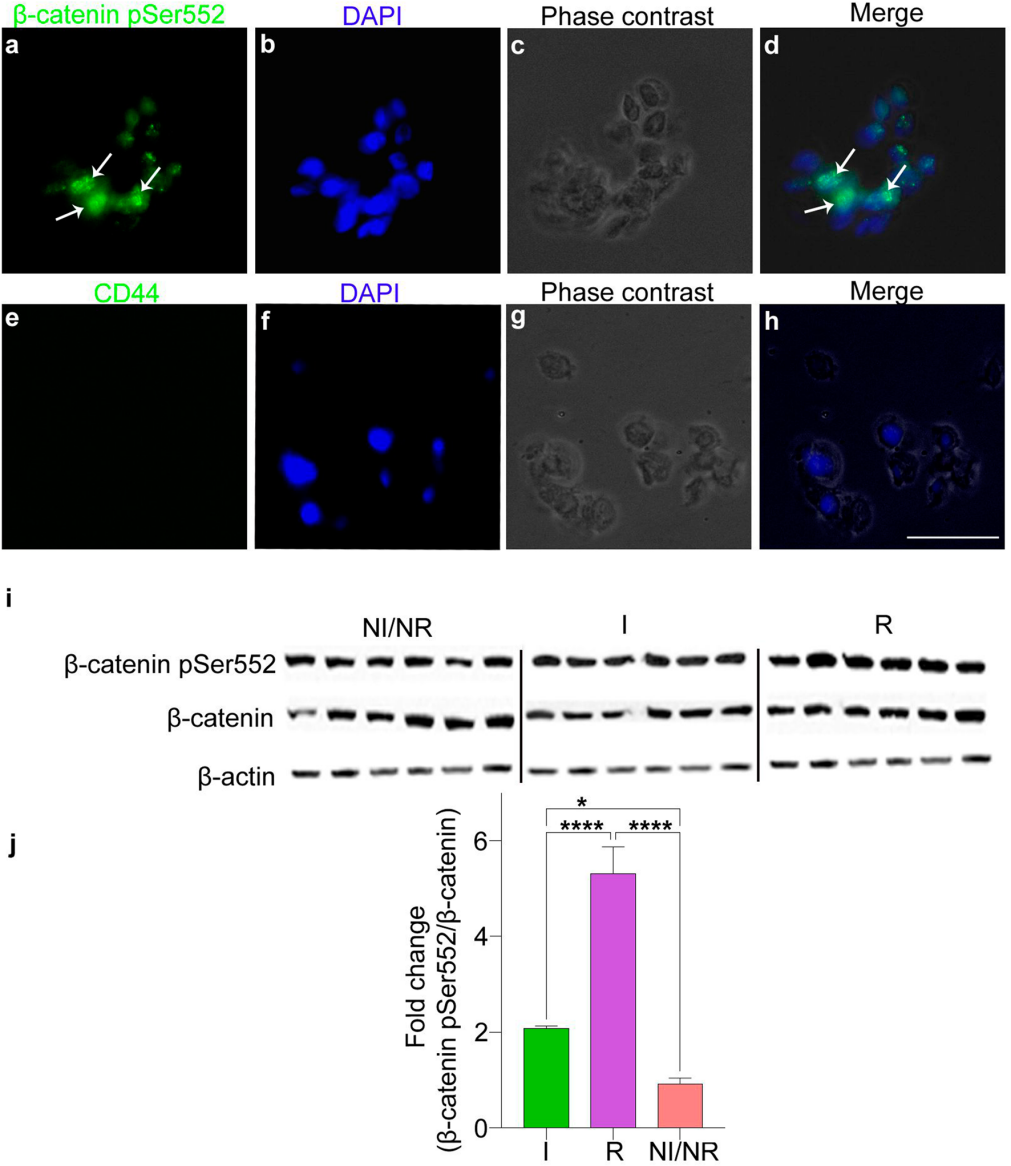
Recurrent NF-PitNETs exhibit up-regulation of β-catenin pSer552. Immunofluorescence reveals that β-catenin pSer552 is predominantly present in the nucleus (green, white arrows in **a**, **d**) localised with DAPI (nuclear blue staining). Cultured NF-PitNET cells were negative for the monocyte marker CD44 which confirms the absence of monocytes in the culture. Images are representative of n = 3 recurrent NF-PitNET cultured tumours. **i**, **j** Western blot revealed increased phosphorylation of β-catenin pSer552 in recurrent compared to non-recurrent NF-PitNETs. **i** Representative image of a western blot of 6 non-invasive/non-recurrent (NI/NR) NF-PitNETs compared to 6 Invasive (I) and 6 recurrent (R) NF-PitNETs immunoblotted against β-catenin pSer552 and total β-catenin. **j** Quantification of the ratio between β-catenin pSer552 and total β-catenin showed a 5.7fold increase in β-catenin pSer552 in recurrent and 2.3fold increase in invasive NFPTs group compared to non-recurrent/non-invasive group. Data is represented as Mean ± SEM. Statistical significant of *p < 0.05, ****p < 0.0001 using two-tailed unpaired Student’s *t*-test. The western blot image is a representative image of 3 independent experiments. Scale bar in H represents 100 µm

To validate our MS results, which indicated that β-catenin pSer552 was upregulated in recurrent NF-PitNETs, we assessed β-catenin pSer552 protein expression by IHC using an antibody against pSer552 β-catenin. IHC was performed in a large cohort of NF-PitNET patients using tissue microarrays. Among these 200 patients, 44 patients had recurrent events (mean follow-up of 10 years, SD ± 5.4). Out of these 44 patients demonstrating progression of disease, 24 underwent a second surgery alone, 10 underwent a second surgery followed by radiation therapy, and 10 received only radiation therapy following the first surgery. In our cohort, tumours were not overtly over-proliferative with only 3 tumours exhibiting a Ki-67 > 3% and 8 tumours were positive for p53. Positive staining for β-catenin pSer552 was observed in both recurrent and invasive NF-PitNETs (Fig. 4). Negative staining of β-catenin pSer552 was found in normal pituitary, and all somatotropinomas (n = 50) (Fig. 4a & e). We then analysed the expression of β-catenin pSer552 on recurrent NF-PitNETs by immunofluorescence in in vitro and identified nuclear positivity of β-catenin pSer552 within tumour cells (Fig. 5a–h). Our results are in line with in vitro studies where phosphorylation of β-catenin at position Ser552 leads to its stabilisation and nuclear localisation [37, 38]. We next validated our phosphoproteomic results using western blotting to quantify amounts of β-catenin pSer552 in non-recurrent/non-invasive tumours comparing invasive and recurrent NF-PitNETs subgroups (Fig. 5i-j). Quantification of immunoblots revealed a 5.7fold increase of phosphorylated β-catenin pSer552 in recurrent (*p* < 0.0001) and 2.3 fold increase in invasive (*p* = 0.04)  NF-PitNETs compared to non-recurrent/non-invasive NF-PitNETs (Fig. 5j). Taken together, the IHC, the immunofluorescence staining and the western blot quantifications of β-catenin pSer552 validate our MS phosphoproteomic results indicating that β-catenin is indeed more phosphorylated at the Ser552 in recurrent group of NF-PitNETs compared to other NF-PitNETs disease subgroups.

**Fig. 6:**
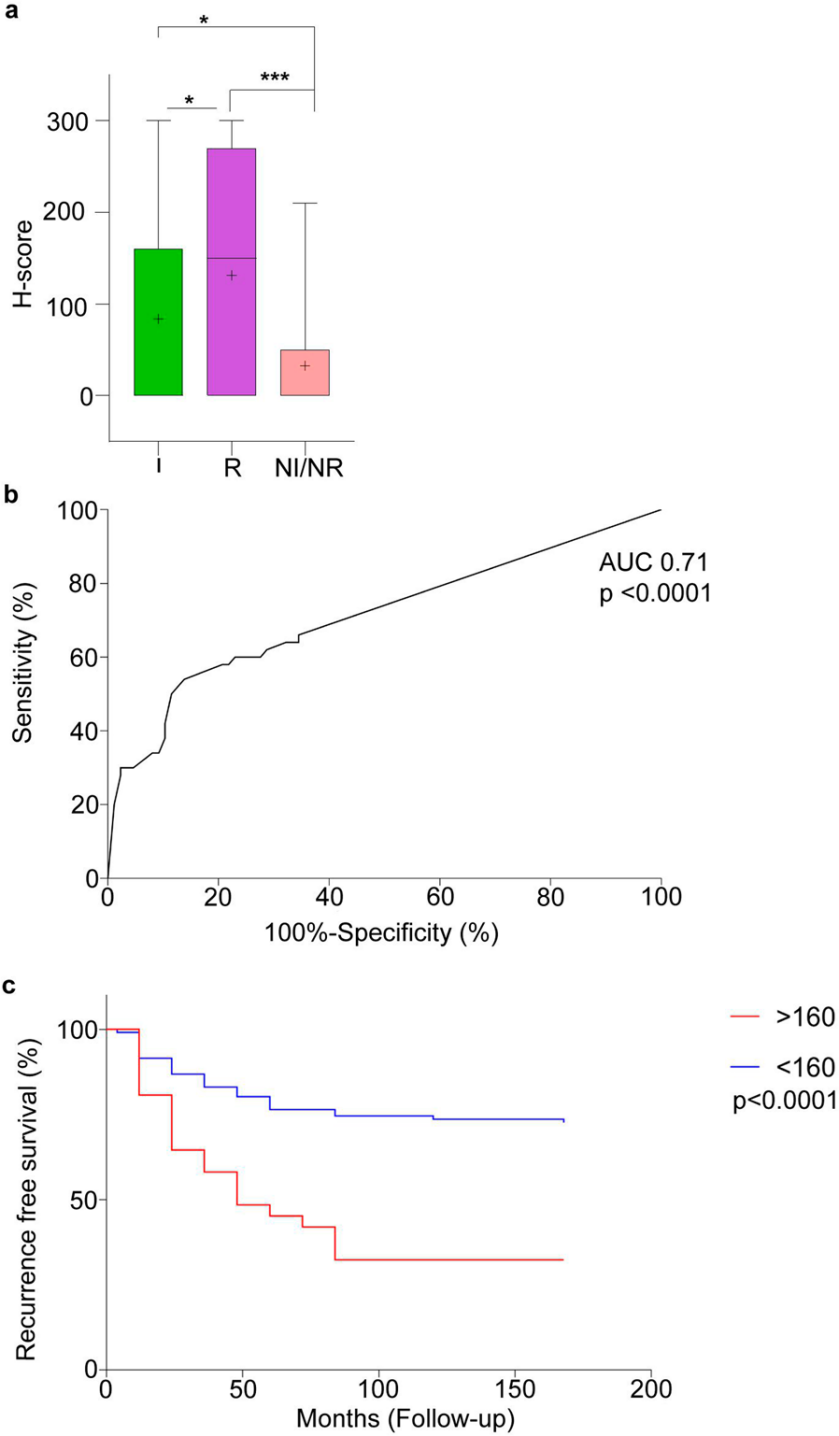
The H-score of β-catenin pSer552 correlates with the recurrence free survival of NF-PitNETs. **a** H-score of immunohistochemistry of β-catenin pSer552 shows significant increase in phosphorylation in recurrent (R) NF-PitNETs as compared to invasive (I) and non-invasive/non-recurrent (NI/NR) NF-PitNETs. **b** Receiver operating characteristic (ROC) curve for the prognosis of recurrent NF-PitNETs. ROC was performed for H-score of recurrent (n = 44) and non-recurrent (n = 156) NF-PitNETs. Area under the curve (AUC) 0.710 (*p* < 0.001) indicates that β-catenin pSer552 can distinguish between recurrent and non-recurrent NF-PitNETs. **c** Kaplan–Meier recurrence-free survival of the 200 NF-PitNETs patients after surgery according to the H-score cut-off level of 160 of β-catenin pSer552. β-catenin pSer552 H-score level > 160 was found to be an independent predictor of tumour NF-PitNETs recurrence (Logrank (Mantel-Cox test) *p* < 0.0001)

We then analysed if the IHC score (H-score) for β-catenin pSer552 could correlate with tumour characteristics such as: tumour recurrence, invasion, suprasellar, parasellar, infrasellar extension, maximum tumour diameter and tumour volume. Quantification of IHC using H-score revealed statistically significant over-expression of β-catenin pSer552 in recurrent (*p* < 0.0001) and invasive (*p* = 0.01) NF-PitNETs compared to the NI/NR tumour subgroup (Fig. 6a, Additional File 1: Table S4). β-catenin pSer552 was found significantly (*p* = 0.01) over-expressed in invasive tumours (Knosp grade 3–4) compared to NI/NR. β-catenin pSer552 expression correlated with suprasellar extension (*p* = 0.02, Additional File 1: Table S4), while infrasellar extension did not showed any association (*p* = 0.48). Maximum tumour diameter (r =  − 0.04, *p* = 0.638) and tumour volume (r = 0.10, *p* = 0.23) were not correlated with the H-score of β-catenin pSer552 in our cohort (Additional File 1: Table S5). Taken together, our data indicates that the phosphorylation status of β-catenin at the Ser552 correlates with the recurrent (r = 0.36, *p* < 0.0001) and also with invasive (r = 0.19, *p* = 0.02) disease subgroup of NF-PitNETs.

To assess if β-catenin pSer552 may function as a prognostic marker, we performed receiver operating characteristics (ROC) curve analysis to find the optimal cut-off value of β-catenin pSer552 H-score in patients who had recurrence (n = 44) or non-recurrence (n = 156) (Additional File 1: Table S6). Although H-score demonstrated statistical significance (*p* < 0.0001) in ROC analysis (Fig. 6b), lower limit of 95% CI of AUC is rather poor (0.613). At the same time and H-score of 160 was confirmed to be optimal cut-off value based on maximum sum of sensitivity and specificity. We then assessed if the β-catenin pSer552’s H-score correlates with the recurrence free survival using Kaplan–Meier survival curves. We found a strong statistical correlation between the recurrence free survival and the nuclear positive staining of β-catenin pSer552 (Fig. 6c, *p* < 0.0001).

### In silico enrichment motive analyses identifies PKA as kinase in recurrent NF-PitNETs

**Fig. 7:**
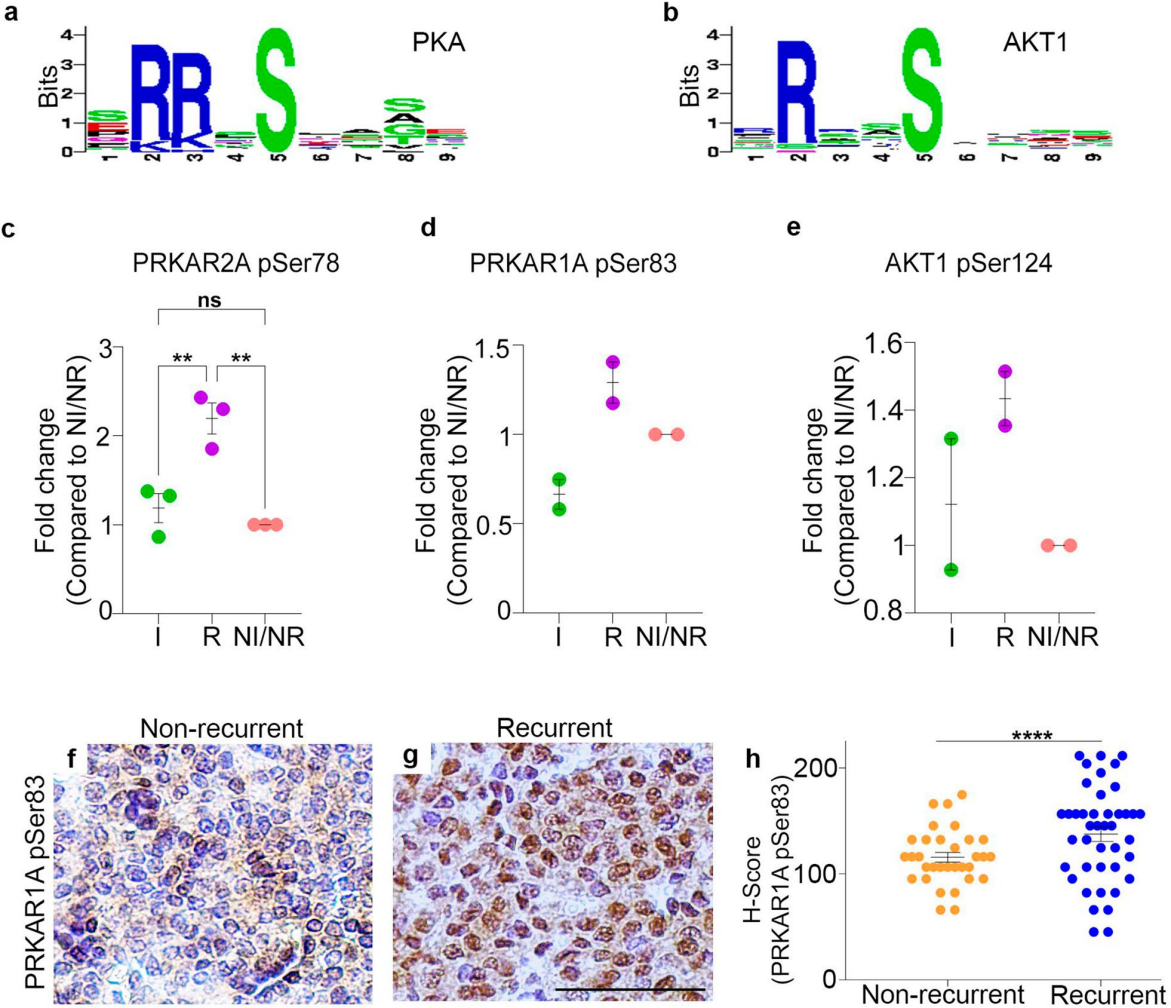
Protein kinase A (PKA) and AKT1 as hyperphosphorylated in recurrent NF-PitNETs. **a**, **b** Motif enrichment analysis using Motif-x identified the kinases up-regulated in recurrent  NF-PitNETs. **c**–**e** Ms quantitation of PRKAR2A pSer78, PRKAR1A pSer83, and AKT pSer124 showed increased phosphorylation in recurrent NF-PitNETs. **c** Increased phosphorylation of PRKAR2A pSer78, a regulatory subunit of PKA was statistically significant. Immunohistochemistry against PRKAR1A pSer83 in non-recurrent (**f**) and recurrent (**g**) tumours showed upregulation of PRKAR1A pSer 83 in NF-PitNETs recurrent tumours. **h** Quantification of H-score of non-recurrent **f** and recurrent **g** tumours revealed hyperphosphorylation of PRKAR1A pSer83 in recurrent NF-PitNETs. Two-tailed unpaired Student’s *t*-test was performed. **p < 0.001, ****p < 0.0001. Scale bar represents 50 μm

To identify which kinases were responsible for the difference in phosphorylation among NF-PitNET subgroups we use Motif-x in silico kinase enrichment analyses. In line with other studies [37, 39], we identified protein kinase A (PKA) and AKT1 as probable kinases involved in phosphorylation differences of the NF-PitNET recurrent group (Fig. 7a & b). Mass-spectrometry data identified the subunits of PKA: PRKAR2A pSer78 and PRKAR1A pSer83 as well as AKT1 pSer124 as being more phosphorylated in recurrent NF-PitNET group compared to invasive and NI/NR (Fig. 7c–e). We also identify increased phosphorylation of PRKAR1A pSer83 in recurrent group of NF-PitNET (Fig. 7g). IHC validation using an antibody against PRKAR1A pSer83 revealed higher number of PRKAR1A pSer83 positive nuclei in recurrent compared to non-recurrent NF-PitNETs. We then utilised the H-score for PRKAR1A pSer83 and identified statistically significant (*p* < 0.0001) fold change increase of PRKAR1A pSer83 in recurrent subgroup compared non-recurrent NF-PitNETs. Hence our result suggests that PRKAR2A pSer78 and or PRKAR1A pSer83 subunit of PKA may mediate some of the phosphorylation events observed in the recurrent NF-PitNET subgroup.

## Discussion

In this study we have utilised LC–MS/MS to characterise the phosphopeptide signature of NF-PitNETs as means to identify biomarkers of tumour recurrence at first surgery. Phosphoproteomic studies of normal pituitary gland [40, 41] and invasive NF-PitNETs [42] have been previously reported. Some of the phosphopeptides identified in normal pituitary studies, such as somatotropin at S136 and S174; 60S acidic ribosomal protein P2 pS102 and pS307; secretogranin1 pS149, pS405; cAMP- Dependent Protein Kinase Type II-Alpha Regulatory Chain pS78 have also been identified in our study as being more phosphorylated in R and I subgroups compared to NI/NR and hence deserve further studies. Work by Liu et al. comparing phosphoproteome of invasive to non-invasive NF-PitNETs reveals that 80% of the phosphosites are also present in our invasive subgroup of NF-PitNETs highlighting the robustness of our study [42]. However, previous phosphoproteomic studies lack validation of their data sets by IHC or western blots in cohorts of patients. Of novelty, our study incorporates the recurrent (R) disease subgroup and reveals differential phosphorylation events in the recurrent NF-PitNETs followed by validation in a large NF-PitNETs cohort. Globally, we identified a higher degree of phosphorylation events in NF-PitNETs recurrent subgroup compared to invasive. Increase in phosphosites in the recurrent NF-PitNETs subgroup may represent higher kinase activity leading to more aggressive tumours. In fact, higher level of phosphorylation activity has been found in several cancers [43, 44] and it has been linked to poor prognosis [43, 45, 46].

From the identified peptides, β-catenin is of particular importance as it is the central component of the Wnt/β-catenin pathway shown to have major roles in embryonic development, stem cell homeostasis and to drive colon, prostate, melanoma and pancreatic tumour formation [47, 48]. Moreover, the Wnt/β-catenin pathway is a key regulator of pituitary development, terminal differentiation and maintenance of pituitary stem cells [49–52]. Somatic mutations in exon 3 of β-catenin in pituitary progenitors/stem cells result in adamantinomatous craniopharyngioma [53–55]. Although, several reports have linked dysregulation of the Wnt/β-catenin pathways with PitNETs [35, 56–58], sanger and whole exome sequencing studies have failed to identify somatic or germline mutations in components Wnt/β-catenin pathway [59–62]. This suggest that posttranslational modifications, such as an increase in phosphorylation of β-catenin could result in upregulation of Wnt pathway in NF-PitNET disease. Indeed, in this study we show increased phosphorylation of β-catenin at Ser552 in recurrent and invasive NF-PitNET’s disease subgroups. H-score of β-catenin pSer552 correlates with recurrence free survival in a cohort of 200 NF-PitNET patients. We show that β-catenin pSer552 exhibits nuclear positivity in recurrent NF-PitNETs both in in vitro culture and by IHC. In the Wnt/β-catenin canonical pathway, phosphorylation of β-catenin can occur at positions Ser45, Ser33, Ser37, and Thr41 by a destruction complex formed by GSK3-β, CK1 together with scaffolding protein Axin2 and APC, leading to β-catenin proteasomal degradation and in turn inhibition of the Wnt pathway [63, 64]. However, β-catenin has also been shown to be phosphorylated in Ser552 and Ser675 independent of the Wnt-canonical pathway, leading to β-catenin stabilisation, translocation to the nucleus and transcriptional activation of Wnt downstream targets genes [38, 39]. Our in silico motif analysis is in line with previous studies in which β-catenin pSer552 has been shown to be a target of both PKA and AKT1 [37–39, 65, 66]. AKT1 has been shown to directly phosphorylate β-catenin at the Ser552 leading to activation of Wnt pathway [37]. However, the site identified in our study, AKT1 pSer124, does not activate AKT1 [67], making it a less likely candidate for our follow up study. We found that the regulatory subunits of PKA: PRKAR2A pSer78 and PRKAR1A pSer83, were phosphorylated in recurrent NF-PitNETs. Although we could not validate PRKAR2A pSer78 due to lack of available specific antibody, future work with anti-PRKAR2A pSer78 specific antibody is warranted. IHC against PRKAR1A pSer83, showed strong correlation with the recurrent subgroup of NF-PitNETs. In this manuscript, we do not provide functional assays to demonstrate that PKA directly phosphorylates β-catenin at Ser552 in NF-PitNETs. Future work will be required to establish the functional role of PRKAR2A pSer78 and PRKAR1A pSer83 in overall PKA activity on β-catenin and its impact on activation of Wnt target genes in recurrent NF-PitNETs.

Tumour recurrence is a major factor of comorbidity in NF-PitNETs leading to poor clinical outcomes [12]. Hence, being able to identify possible markers that correlate with tumour recurrence at first surgery may have prognostic value, particularly in patients likely to recur, as they could be kept under closer follow-up. Our study, using a large cohort of NF-PitNEts, has identified that β-catenin pSer552 status correlates both with recurrence and invasion of NF-PitNETs. Validation of our results in external cohorts is required to strengthen the value of β-catenin pSer552 as possible biomarker of NF-PitNET recurrence at first surgery.

## Supplementary Information

**Additional file 1:** Supplementary Tables.

**Additional file 2: Fig. S1.** Classification of the NF-PitNET’s subgroups based on the expression of transcription factors.** a**–**d** Immunohistochemistry against PIT1 (**a**), TPIT (**b**), and SF1 (**c**). Tumours negative for all three PIT1, TPIT, and SF1 were classified as null cell tumours (**d**). Positive immunostaining is shown by brown colour marked by black arrows. Scale bar in H represents 100 µm.

**Additional File 3: Fig. S2**. Experimental workflow of phosphoproteomic analysis of NF-PitNETs. Patients were divided into three groups: non-invasive/non-recurrent (NI/NR), invasive (I), and recurrent (R). For quantification peptides were labelled with tandem mass tags (TMT). Phosphopeptide enrichment was done by titanium dioxide (TiO2) and fractionated by basic reverse phase liquid chromatography (bRPLC) prior to LC–MS/MS analysis on two different mass spectrometrs, Orbitrap Velos and Orbitarp Fusion Tribrid mass spectrometer (ThermoScientific). Peptides identified in triplicates were further used for in-silico functional analysis and validation on large cohort (n = 200) by immunohistochemistry on tissue microarray (TMA).

**Additional File 4: Fig. S3**. Differential phosphorylation of Ser, Thr, and Tyr in recurrent NF-PitNETs and reproducibility of data. **a**–**c** Fisher’s test shows significantly high number of hyper and hypo phosphorylated Ser (**a**, p< 0.0001) and Thr (**b**, *p* = 0.01) phosphopeptides in R as compared to I. **c** No difference in number of phosphotyrosine was found. **d**, **e** Pearson’s correlation coefficient for invasive (**d**, r = 0.78) and recurrent (**e**, r = 0.73) indicate strong reproducibility among replicates (*p* < 0.0001). *I* Invasive; *R* Recurrent; *Ser* Serine; *Thr* Threonine; *Tyr* Tyrosine.

**Additional File 5: Fig. S4**. Increased upregulated phosphopeptides in the recurrent NF-PitNET subgroup.** a**,** b** Graphical representation showing the number of phosphopeptides with the Log2-fold change in the various NF-PitNET groups with red lines indicating hypo- and hyper-phosphorylated peptides. c Principle component (PC) analysis of NF-PitNET phosphoproteome reveal disease subgroup segregation and replicates group together. Singular value decomposition (SVD) with imputation of fold change of phosphopeptides of each NF-PitNET subgroup is used to calculate principal components. X and Y axis show principal component 1(PC1) and principal component 2 (PC2) that explain 63.3% and 82.8% of the total variance, respectively. Each grey dots represents an NF-PitNET subgroup. Abbreviations: NI/NR, non-invasive/non-recurrent; *I*, Invasive; *R*, Recurrent; *PC* Principle component. Green colour represents invasive (I) subgroups while blue colour represents recurrent (R).

**Additional File 6: Fig. S5**. Gene ontology (GO) pathway analyses showing enriched (red) or depleted (green) hyperphosphorylated proteins in R NF-PitNET. **a**–**d** Phosphoproteins exclusively overphosphorylated in recurrent PitNETs were used for gene ontology analysis using FunRich (version 3.1.3) software. X axis represents fold change enrichment of GO categories in recurrent NF-PitNETs as compared to non-invasive/non-recurrent and Y axis represents the GO categories. **a** Cell component analysis showed endoplasmic reticular membrane proteins were most enriched followed by cell cortex, ribosome, nuclear speck, and cytoplasmic microtubules. **b** Graphical representation of molecular function revealed proteins with ATPase activity were most enriched while caspase activator proteins were most depleted in recurrent NF-PitNETs. c Graphical representation of biological process showed proteins involved in cell proliferation, regulation of cell proliferation and migration were most enriched. d Graphical representation of biological pathways showed glypican-3 signalling and overall high enrichment of Wnt signalling and regulation of nuclear β-catenin signalling as the most enriched pathways in recurrent NF-PitNETs. *R* Recurrence; *GO* Gene ontology

**Additional File 7: Fig. S6**. Protein-protein interaction network of phosphoproteins exclusively overphosphorylated in recurrent NFPTs showed upregulation of β-catenin signalling. Phosphoproteins only overphosphorylated in recurrent subgroup (R) were mapped using FunRich (version 3.1.3) software. Each red node represents a hyperphosphorylated protein and blue line indicates interactions. EGFR as found to be in the center of interaction hub while β-catenin was found to be part of most of the upregulated pathways. Blue arrows indicate β-catenin and MYH9, which are significantly hyperphosphorylated in R. Nodes with green circles represents proteins involved in regulation of nuclear β-catenin signaling, while nodes with yellow circles represents proteins involved in developmental pathways. PSMD2 and PSMA3 (indicated by black arrows) represents proteins involved in cell cycle regulation.
