# Supplementary material for: Phosphorylation of β-catenin at Serine552 correlates with invasion and recurrence of non-functioning pituitary neuroendocrine tumours
Source: Acta Neuropathol Commun. 2022 Sep 16;10:138. doi: 10.1186/s40478-022-01441-5 (PMC9482208; doi:10.1186/s40478-022-01441-5)
Supplement: Supplementary file 1 — Additional file 1: Supplementary Tables. [file 40478_2022_1441_MOESM1_ESM.docx]

**Suppelementary Tables**

**Supplementary Table 1**: Clinical and histopathological variables of NF-PitNET patients for discovery phase.

| **Case** | **Age** | **TV** | **KG** | **Invasive intra- operative**  **impression** | **Trouillas**  **Tumour Grade** | **Histopathological**  **Invasion** | | | **Immunohistochemistry** | | | | | | **Transcription factors** | | |
| --- | --- | --- | --- | --- | --- | --- | --- | --- | --- | --- | --- | --- | --- | --- | --- | --- | --- |
|  | **Yr** | **(cm^3^)** |  |  |  | **B** | **M** | **D** | **TSH** | **PRL** | **LH** | **ACTH** | **GH** | **FSH** | **SF1** | **PIT1** | **TPIT** |
| **Non-invasive/non-recurrent (NI/NR)** | | | | | | | | | | | | | | | | | |
| 1 | 45 | 27.9 | 2 | No | 1a | 0 | 0 | 0 | 0 | 0 | 0 | 0 | 0 | 0 | 1 | 0 | 0 |
| 2 | 32 | 44 | 0 | No | 1a | 0 | 0 | 0 | 0 | 0 | 1 | 0 | 0 | 0 | 1 | 0 | 0 |
| 3 | 60 | 71.1 | 2 | No | 1a | 0 | 0 | 0 | 0 | 1 | 0 | 0 | 0 | 0 | 0 | 1 | 0 |
| 4 | 65 | 67.6 | 0 | No | 1a | 0 | 0 | 0 | 0 | 0 | 0 | 0 | 0 | 0 | 1 | 0 | 0 |
| 5 | 57 | 31.5 | 2 | No | 1a | 0 | 0 | 0 | 0 | 0 | 0 | 0 | 0 | 0 | 1 | 0 | 0 |
| **Invasive (I)** | | | | | | | | | | | | | | | | | |
| 6 | 57 | 34.2 | 4 | Yes | 2a | 1 | 1 | 1 | 0 | 0 | 0 | 1 | 0 | 0 | 0 | 0 | 1 |
| 7 | 54 | 58.9 | 4 | Yes | 2a | 1 | 1 | 1 | 0 | 0 | 0 | 0 | 0 | 0 | 1 | 0 | 0 |
| 8 | 48 | 15.3 | 3 | Yes | 2a | 1 | 1 | 1 | 0 | 0 | 0 | 1 | 0 | 0 | 0 | 0 | 1 |
| 9 | 42 | 40 | 3 | Yes | 2a | 0 | 1 | 1 | 0 | 0 | 0 | 0 | 0 | 0 | 1 | 0 | 0 |
| 10 | 42 | 10.1 | 4 | Yes | 2a | 0 | 1 | 1 | 0 | 0 | 0 | 0 | 0 | 0 | 0 | 0 | 0 |
| 11 | 35 | 19.5 | 3 | Yes | 2a | 0 | 0 | 0 | 0 | 0 | 0 | 0 | 0 | 0 | 1 | 0 | 0 |
| 12 | 65 | 10.5 | 3 | Yes | 2a | 0 | 0 | 0 | 0 | 0 | 0 | 0 | 0 | 0 | 0 | 0 | 0 |
| 13 | 62 | 18 | 4 | Yes | 2a | 0 | 0 | 0 | 0 | 0 | 0 | 0 | 0 | 0 | 0 | 0 | 0 |
| 14 | 35 | 13.1 | 4 | Yes | 2a | 0 | 0 | 0 | 0 | 0 | 0 | 0 | 0 | 0 | 1 | 0 | 0 |
| 15 | 28 | 28 | 4 | Yes | 2a | 0 | 0 | 0 | 0 | 0 | 0 | 0 | 0 | 0 | 1 | 0 | 0 |
| **Recurrent (R)** | | | | | | | | | | | | | | | | | |
| 16 | 65 | 77 | 4 | Yes | 2a | 0 | 0 | 1 | 0 | 0 | 0 | 0 | 0 | 0 | 1 | 0 | 0 |
| 17 | 50 | 15.3 | 2 | No | 1a | 0 | 0 | 0 | 0 | 0 | 0 | 0 | 1 | 0 | 0 | 1 | 0 |
| 18 | 35 | 39.6 | 4 | Yes | 2a | 0 | 0 | 0 | 0 | 0 | 0 | 0 | 0 | 0 | 0 | 0 | 0 |
| 19 | 40 | 18 | 3 | No | 1a | 0 | 0 | 0 | 0 | 0 | 0 | 0 | 0 | 0 | 1 | 0 | 0 |
| 20 | 50 | 15.6 | 4 | Yes | 2a | 0 | 0 | 1 | 0 | 0 | 0 | 0 | 1 | 0 | 0 | 1 | 0 |

Cases 1-5 represents non-invasive/non-recurrent (NI/NR), 6-15 represents invasive (I), and 16-20 represent recurrent (R) NF-PitNETs; Yr- yearsTV-Tumour volume, KG-Knosp Grade, B-bone, M-mucosa, D-dura TSH-thyroid stimulating hormone, PRL- prolactin, LH-leutinising hormone, ACTH-adrenocorticotropic hormone, GH-growth hormone, FSH- follicle stimulating hormone; 0=negative, 1=positive.

.

**Supplementary Table 2:** Clinicopathological characteristics of patients of validation cohort.

| **Total number of patients included** | **NF-PitNET (n=200)** |
| --- | --- |
| Gender (Males: Females) | 122 (61.1%): 78 (38.8%) |
| Mean Age (years) | 43.1 (±12.6) |
| Complete resection | 76(38%) |
| Residual Lesion | 82(41%) |
| Underwent second surgery | 23(11.5%) |
| Post-op RT/gamma knife | 21(10.5%) |
| **Immunohistochemistry** | |
| Goanadotroph tumour (SF1-lineage PitNETs) | 147(73.5%) |
| Corticotroph tumour  (TPIT-lineage PitNETs) | 15(7.5%) |
| Immature PIT1-lineage tumour | 9 (4.5%) |
| PitNETs with no distinct cell lineage | |
| Null cell tumour | 16(8%) |
| Plurihormonal tumour | 13(6.5%) |
| **Extension** | |
| Supra sellar extension | 116 |
| Parasellar extension | 46 |
| Infrasellar extension | 17 |
| Intrasellar (within sella region) | 21 |
| **Knosp Grade** |  |
| 0 | 46 |
| 1&2 | 70 |
| 3&4 | 84 |
| **Histopathological invasion** |  |
| Present | 84 |
| Absent | 116 |
| **Trouillas Classification** |  |
| 1a | 116 |
| 1b | 0 |
| 2a | 82 |
| 2b | 2 |

**Supplementary Table 3:** List of phosphopeptides identified in triplicates

|  | | | **Fold Change**  **(compared to NI/NR)** | |
| --- | --- | --- | --- | --- |
| **S. No.** | **Protein** | **Full Name** | **I** | **R** |
| 1 | ABCF1 | ATP Binding Cassette Subfamily F Member 1 | 0.98 | 2.07 |
| 2 | AHNAK | AHNAK Nucleoprotein | 0.13 | 0.45 |
| 3 | AHSG | Alpha 2-HS Glycoprotein | 1.18 | 3.64 |
| 4 | ALAD | Aminolevulinate Dehydratase | 0.97 | 0.85 |
| 5 | ANKIB1 | Ankyrin Repeat And IBR Domain Containing 1 | 0.84 | 0.53 |
| 6 | BAG6 | BAG Cochaperone 6 | 1.14 | 0.97 |
| 7 | BIN1 | Bridging Integrator 1 | 1.03 | 1.20 |
| 8 | BLOC1S3 | Biogenesis Of Lysosomal Organelles Complex 1 Subunit 3 | 1.44 | 1.21 |
| 9 | C11orf58 | Chromosome 11 Open Reading Frame 58 | 1.88 | 1.25 |
| 10 | C9orf142 | Chromosome 9 Open Reading Frame 142 | 0.73 | 0.74 |
| 11 | CAMK2B | Calcium/Calmodulin Dependent Protein Kinase II Beta | 1.03 | 0.79 |
| 12 | CANX | Calnexin | 1.05 | 2.02 |
| 13 | CBX3 | Chromobox 3 | 0.94 | 2.21 |
| 14 | CDS2 | CDP-Diacylglycerol Synthase 2 | 1.15 | 1.70 |
| 15 | CHGB | Chromogranin B | 5.50 | 3.99 |
| 16 | CHGB | Chromogranin B | 0.89 | 2.15 |
| 17 | CHGB | Chromogranin B | 3.11 | 0.61 |
| 18 | CROCC | Ciliary Rootlet Coiled-Coil, Rootletin | 1.19 | 0.92 |
| 19 | CTNNB1 | Catenin Beta 1 | 1.10 | 2.02 |
| 20 | CTR9 | CTR9 Homolog, Paf1/RNA Polymerase II Complex Component | 0.74 | 0.23 |
| 21 | CTTN | Cortactin | 0.84 | 0.83 |
| 22 | DDX24 | DEAD-Box Helicase 24 | 1.06 | 1.73 |
| 23 | DDX51 | DEAD-Box Helicase 51 | 0.76 | 1.01 |
| 24 | DMTN | Dematin Actin Binding Protein | 0.74 | 2.56 |
| 25 | DYNC1LI2 | Dynein Cytoplasmic 1 Light Intermediate Chain 2 | 1.12 | 0.66 |
| 26 | EIF2B5 | Eukaryotic Translation Initiation Factor 2B Subunit Epsilon | 1.03 | 0.91 |
| 27 | EPN2 | Epsin 2 | 0.84 | 0.73 |
| 28 | EPRS | Glutamyl-Prolyl-TRNA Synthetase 1 | 0.97 | 0.69 |
| 29 | FAM169A | Family With Sequence Similarity 169 Member A | 1.52 | 1.26 |
| 30 | FAM169A | Family With Sequence Similarity 169 Member A | 1.71 | 1.28 |
| 31 | FIP1L1 | Factor Interacting With PAPOLA And CPSF1 | 0.86 | 0.71 |
| 32 | FOXK1 | Forkhead Box K1 | 1.01 | 0.91 |
| 33 | HDAC1 | Histone Deacetylase 1 | 0.82 | 0.82 |
| 34 | HDAC2 | Histone Deacetylase 2 | 0.78 | 0.49 |
| 35 | HIRIP3 | HIRA Interacting Protein 3 | 0.75 | 0.49 |
| 36 | HSF1 | Heat Shock Transcription Factor 1 | 1.01 | 1.44 |
| 37 | HTT | Huntingtin | 0.93 | 1.22 |
| 38 | IFI16 | Interferon Gamma Inducible Protein 16 | 0.63 | 0.64 |
| 39 | ITIH2 | Inter-Alpha-Trypsin Inhibitor Heavy Chain 2 | 1.06 | 3.71 |
| 40 | IWS1 | Interacts With SUPT6H, CTD Assembly Factor 1 | 0.99 | 0.63 |
| 41 | LRRC47 | Leucine Rich Repeat Containing 47 | 1.39 | 2.64 |
| 42 | MAP1S | Microtubule Associated Protein 1S | 0.99 | 0.62 |
| 43 | MARCKS | Myristoylated Alanine Rich Protein Kinase C Substrate | 1.56 | 3.14 |
| 44 | MBD2 | Methyl-CpG Binding Domain Protein 2 | 0.99 | 0.93 |
| 45 | MYH9 | Myosin Heavy Chain 9 | 1.12 | 2.58 |
| 46 | NAP1L4 | Nucleosome Assembly Protein 1 Like 4 | 1.41 | 1.90 |
| 47 | NCL | Nucleolin | 1.00 | 1.41 |
| 48 | NPDC1 | Neural Proliferation, Differentiation And Control 1 | 1.82 | 1.11 |
| 49 | NUCKS1 | Nuclear Casein Kinase And Cyclin Dependent Kinase Substrate 1 | 2.16 | 2.31 |
| 50 | NUCKS1 | Nuclear Casein Kinase And Cyclin Dependent Kinase Substrate 2 | 1.65 | 3.21 |
| 51 | NUCKS1 | Nuclear Casein Kinase And Cyclin Dependent Kinase Substrate 3 | 0.45 | 1.24 |
| 52 | OSBP | Oxysterol Binding Protein | 1.03 | 0.78 |
| 53 | PBDC1 | Polysaccharide Biosynthesis Domain Containing 1 | 1.17 | 1.66 |
| 54 | PDAP1 | PDGFA Associated Protein 1 | 1.26 | 2.59 |
| 55 | PGM2L1 | Phosphoglucomutase 2 Like 1 | 1.65 | 1.38 |
| 56 | PHC3 | Polyhomeotic Homolog 3 | 1.00 | 0.90 |
| 57 | PHIP | Pleckstrin Homology Domain Interacting Protein | 1.02 | 1.01 |
| 58 | PI4K2A | Phosphatidylinositol 4-Kinase Type 2 Alpha | 1.07 | 1.08 |
| 59 | PLCL2 | Phospholipase C Like 2 | 0.99 | 0.87 |
| 60 | PLEKHA6 | Pleckstrin Homology Domain Containing A6 | 1.85 | 2.27 |
| 61 | PNKP | Polynucleotide Kinase 3'-Phosphatase | 2.02 | 1.22 |
| 62 | PNN | Pinin, Desmosome Associated Protein | 1.37 | 1.93 |
| 63 | POMC | Proopiomelanocortin | 18.23 | 2.80 |
| 64 | PPIG | Peptidylprolyl Isomerase G | 0.87 | 0.69 |
| 65 | PRKAR2A | Protein Kinase CAMP-Dependent Type II Regulatory Subunit Alpha | 1.19 | 1.94 |
| 66 | PRKRA | Protein Activator Of Interferon Induced Protein Kinase EIF2AK2 | 1.21 | 1.99 |
| 67 | PRPF40A | Pre-MRNA Processing Factor 40 Homolog A | 0.97 | 0.83 |
| 68 | PRPF4B | Pre-MRNA Processing Factor 4B | 0.88 | 0.51 |
| 69 | PWP1 | PWP1 Homolog, Endonuclein | 0.77 | 1.02 |
| 70 | RAI1 | Retinoic Acid Induced 1 | 1.24 | 0.82 |
| 71 | RALY | RALY Heterogeneous Nuclear Ribonucleoprotein | 0.86 | 0.61 |
| 72 | RBM25 | RNA Binding Motif Protein 25 | 1.75 | 4.39 |
| 73 | RCSD1 | RCSD Domain Containing 1 | 0.72 | 1.27 |
| 74 | RING1 | Ring Finger Protein 1 | 1.45 | 1.43 |
| 75 | RNF20 | Ring Finger Protein 20 | 0.89 | 1.10 |
| 76 | RPLP0 | Ribosomal Protein Lateral Stalk Subunit P0 | 1.30 | 2.15 |
| 77 | RPLP2 | Ribosomal Protein Lateral Stalk Subunit P2 | 2.17 | 4.61 |
| 78 | SCAF1 | SR-Related CTD Associated Factor 1 | 1.02 | 1.12 |
| 79 | SCAF1 | SR-Related CTD Associated Factor 2 | 1.11 | 0.69 |
| 80 | SGTA | Small Glutamine Rich Tetratricopeptide Repeat Co-Chaperone Alpha | 0.90 | 1.99 |
| 81 | SLC2A13 | Solute Carrier Family 2 Member 13 | 0.87 | 0.97 |
| 82 | SLC8A2 | Solute Carrier Family 8 Member A2 | 0.95 | 0.61 |
| 83 | SMARCC2 | SWI/SNF Related, Matrix Associated, Actin Dependent Regulator Of Chromatin Subfamily C Member 2 | 1.06 | 0.99 |
| 84 | SMN2 | Survival Of Motor Neuron 2, Centromeric | 0.94 | 1.20 |
| 85 | SNIP1 | Smad Nuclear Interacting Protein 1 | 0.97 | 0.77 |
| 86 | SNRNP200 | Small Nuclear Ribonucleoprotein U5 Subunit 200 | 1.35 | 0.72 |
| 87 | SNTB2 | Syntrophin Beta 2 | 0.87 | 0.64 |
| 88 | SNW1 | SNW Domain Containing 1 | 0.91 | 0.93 |
| 89 | SPARCL1 | SPARC Like 1 | 1.42 | 0.33 |
| 90 | SPTBN2 | Spectrin Beta, Non-Erythrocytic 2 | 1.49 | 0.75 |
| 91 | SRRM1 | Serine And Arginine Repetitive Matrix 1 | 1.07 | 1.32 |
| 92 | SRSF1 | Serine And Arginine Rich Splicing Factor 1 | 0.78 | 0.60 |
| 93 | SRSF2 | Serine And Arginine Rich Splicing Factor 2 | 0.92 | 0.96 |
| 94 | SRSF6 | Serine And Arginine Rich Splicing Factor 6 | 0.83 | 0.83 |
| 95 | STARD10 | StAR Related Lipid Transfer Domain Containing 10 | 1.34 | 1.05 |
| 96 | TAF3 | TATA-Box Binding Protein Associated Factor 3 | 0.99 | 0.97 |
| 97 | TBC1D10B | TBC1 Domain Family Member 10B | 0.84 | 0.77 |
| 98 | TCEAL3 | Transcription Elongation Factor A Like 3 | 1.22 | 1.94 |
| 99 | TMEM11 | Transmembrane Protein 11 | 1.39 | 0.68 |
| 100 | TMEM245 | Transmembrane Protein 245 | 0.84 | 0.70 |
| 101 | TMX1 | Thioredoxin Related Transmembrane Protein 1 | 1.16 | 2.41 |
| 102 | TOMM70A | Translocase Of Outer Mitochondrial Membrane 70 | 0.88 | 1.15 |
| 103 | TP53BP1 | Tumor Protein P53 Binding Protein 1 | 1.55 | 2.78 |
| 104 | TRA2A | Transformer 2 Alpha Homolog | 1.07 | 0.83 |
| 105 | TRIM28 | Tripartite Motif Containing 28 | 1.54 | 0.90 |
| 106 | TRIM3 | Tripartite Motif Containing 3 | 0.84 | 1.10 |
| 107 | UFL1 | UFM1 Specific Ligase 1 | 1.41 | 2.83 |
| 108 | USP39 | Ubiquitin Specific Peptidase 39 | 1.00 | 1.48 |
| 109 | VIM | Vimentin | 0.39 | 0.30 |
| 110 | XRN2 | 5'-3' Exoribonuclease 2 | 1.15 | 0.75 |

I- invasive, R- recurrence, NI/NR-non-invasive/non-recurrent

**Supplementary Table 4:** Association of β-catenin pSer552 H-score with NF-PitNET tumour characterstics.

|  | | | | **SSE** | | **ISE** | |
| --- | --- | --- | --- | --- | --- | --- | --- |
|  | **NI/NR** | **I** | **R** | **Present** | **Absent** | **Present** | **Absent** |
| **Mean±SEM** | 32±9.2 | 83.7±13.4 | 131.4±17.79 | 89.24±11.69 | 50.6±13.0 | 97.1±31.2 | 74.0±9.3 |
| **Median (IQR)** | 0(0-50) | 0(0-160) | 150 (0-270) | 40 (0-160) | 0 (0-100) | 40 (0-180) | 0 (0-145) |
| **p Value** |  | 0.01* | <0.0001**** | 0.02* | | 0.48 | |

**Supplementary Table 5:** Correlation of β-catenin pSer552 H-score with NF-PitNET tumour diameter and volume.

|  | **Hscore vs. Tumour diameter** | **Hscore vs. Tumour volume** |
| --- | --- | --- |
| **r** | -0.04054 | 0.1015 |
| **95% Confidence Interval** | -0.2069 to 0.1280 | -0.06739 to 0.2647 |
| **p Value** | 0.6381 | 0.2381 |

**Supplementary Table 6:** Result of ROC-analysis of β-catenin pSer552 for prediction of recurrence in NF-PitNET.

| **Parameter** | **N** | **AUC** | **Standard**  **Error** | **pValue** | **95% Confidence Limits** | |
| --- | --- | --- | --- | --- | --- | --- |
|  |  |  |  |  | **Lower** | **Upper** |
| H-score | 200 | 0.717 | 0.0475 | <0.0001 | 0.6106 | 0.7979 |

**Supplementary Table 7:** Youden’s index and sum of sensitivity and specificity for β-catenin pSer552 for prediction of recurrence in NF-PitNET

| **Cutoff Value** | **Youden Index** | **Sens. + Spec.** |
| --- | --- | --- |
| ≥ 160.00 | 0.3251 | 1.3251 |
| ≥ 140.00 | 0.3221 | 1.3221 |
| ≥ 30.00 | 0.2952 | 1.2952 |
| ≥ 120.00 | 0.2931 | 1.2931 |
| ≥ 60.00 | 0.2926 | 1.2926 |
| ≥ 100.00 | 0.2901 | 1.2901 |
| ≥ 240.00 | 0.2855 | 1.2855 |
| ≥ 70.00 | 0.2841 | 1.2841 |
| ≥ 110.00 | 0.2816 | 1.2816 |
| ≥ 200.00 | 0.2795 | 1.2795 |
| ≥ 95.00 | 0.2786 | 1.2786 |
| ≥ 50.00 | 0.2782 | 1.2782 |
| ≥ 270.00 | 0.277 | 1.277 |
| ≥ 75.00 | 0.2756 | 1.2756 |
| ≥ 40.00 | 0.2752 | 1.2752 |
| ≥ 210.00 | 0.274 | 1.274 |
| ≥ 190.00 | 0.268 | 1.268 |
| ≥ 180.00 | 0.2566 | 1.2566 |
| ≥ 80.00 | 0.2556 | 1.2556 |
| ≥ 300.00 | 0.2085 | 1.2085 |

**Supplementary Table 8**: Definitions of mass spectrometry related terminology

| **S.No.** | **Terminology** | **Definition** |
| --- | --- | --- |
| 1 | Class I Phosphopeptide | Phosphopeptide in which, localisation probability of phosphorylation site is 75% or above. |
| 2 | Ion Score | It is a measure of how well the observed mass spectrum matches the stated peptide. |
| 3 | XCorr | It is a measure of the goodness of fit of experimental peptide fragments to theoretical spectra created*. |

*Jimmy K. Eng, Ashley L. McCormack, and John R. Yates, III; An Approach to Correlate Tandem Mass Spectral Data of Peptides with Amino Acid Sequences in a Protein Database. J. Am. Soc. Mass Spectrom. 1994, 5, 976-989.
