# Supplementary material for: Phosphorylation of β-catenin at Serine552 correlates with invasion and recurrence of non-functioning pituitary neuroendocrine tumours
Source: Acta Neuropathol Commun. 2022 Sep 16;10:138. doi: 10.1186/s40478-022-01441-5 (PMC9482208; doi:10.1186/s40478-022-01441-5)
Supplement: Supplementary file 2 — Additional file 2: Fig. S1. Classification of the NF-PitNET’s subgroups based on the expression of transcription factors. a–d Immunohistochemistry against PIT1 (a), TPIT (b), and SF1 (c). Tumours negative for all three PIT1, TPIT, and SF1 were classified as null cell tumours (d). Positive immunostaining is shown by brown colour marked by black arrows. Scale bar in H represents 100 µm. [file 40478_2022_1441_MOESM2_ESM.pdf]

Supplementary Fig. 1

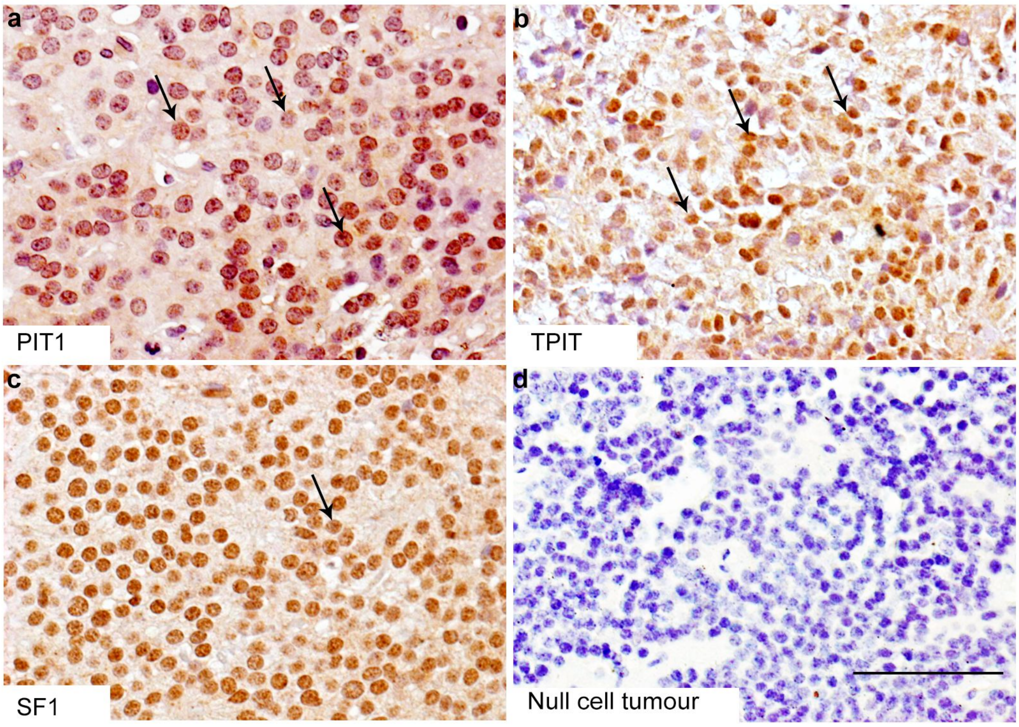

Supplementary Fig.1 Classification of the NF-PitNET's subgroups based on the expression of transcription factors. (a-d) Immunohistochemistry against PIT1 (a), TPIT (b), and SF1 (c). Tumours negative for all three PIT1, TPIT, and SF1 were classified as null cell tumours (d). Positive immunostaining is shown by brown colour marked by black arrows. Scale bar in H represents 100µm.
