# Supplementary material for: Phosphorylation of β-catenin at Serine552 correlates with invasion and recurrence of non-functioning pituitary neuroendocrine tumours
Source: Acta Neuropathol Commun. 2022 Sep 16;10:138. doi: 10.1186/s40478-022-01441-5 (PMC9482208; doi:10.1186/s40478-022-01441-5)
Supplement: Supplementary file 3 — Additional File 3: Fig. S2. Experimental workflow of phosphoproteomic analysis of NF-PitNETs. Patients were divided into three groups: non-invasive/non-recurrent (NI/NR), invasive (I), and recurrent (R). For quantification peptides were labelled with tandem mass tags (TMT). Phosphopeptide enrichment was done by titanium dioxide (TiO2) and fractionated by basic reverse phase liquid chromatography (bRPLC) prior to LC–MS/MS analysis on two different mass spectrometrs, Orbitrap Velos and Orbitarp Fusion Tribrid mass spectrometer (ThermoScientific). Peptides identified in triplicates were further used for in-silico functional analysis and validation on large cohort (n = 200) by immunohistochemistry on tissue microarray (TMA). [file 40478_2022_1441_MOESM3_ESM.pdf]

Supplementary Fig. 2

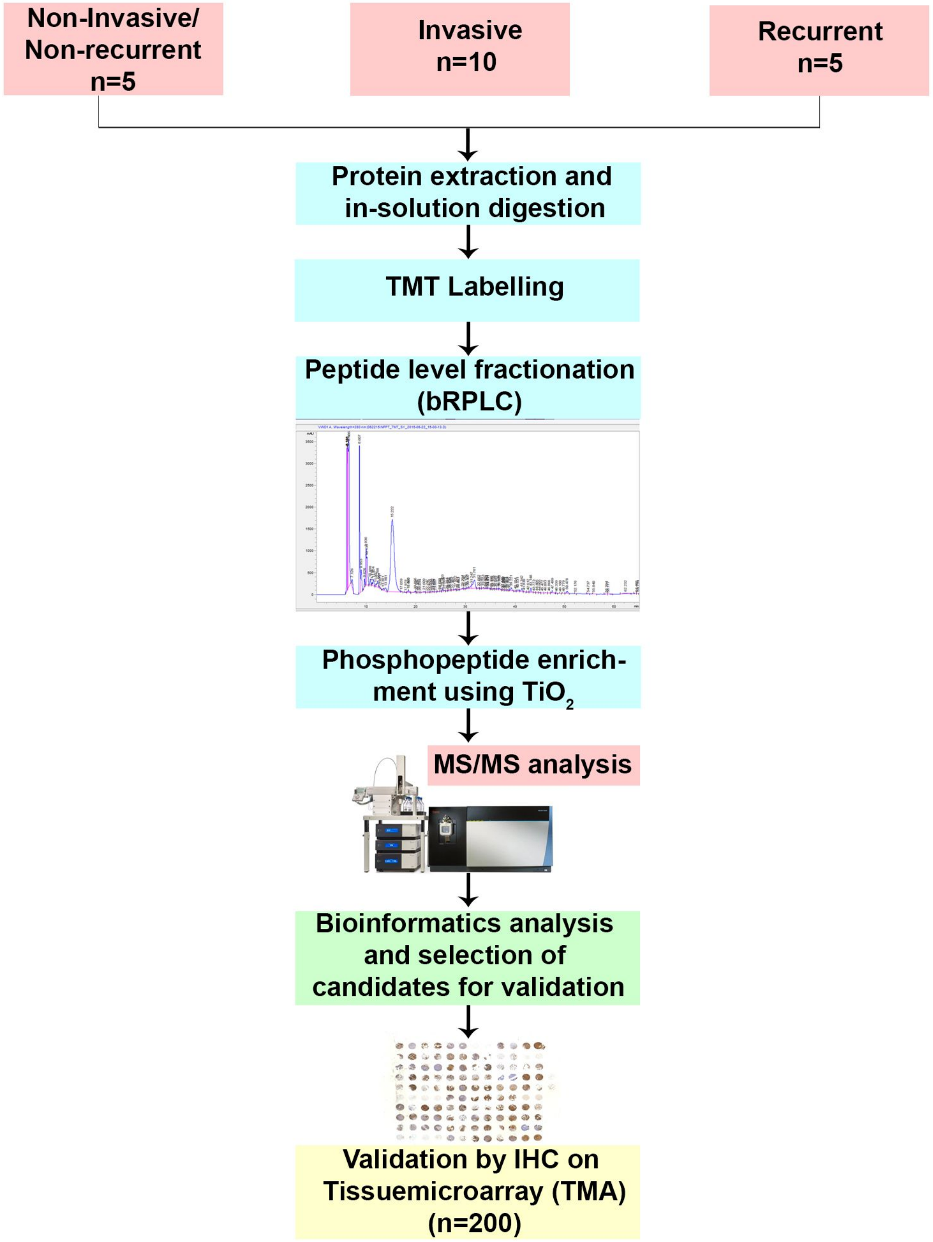

Supplementary Fig. 2 Experimental workflow of phosphoproteomic analysis of NF-PitNETs. Patients were divided into three groups: non-invasive/non-recurrent (NI/NR), invasive (I), and recurrent (R). For quantification peptides were labelled with tandem mass tags (TMT). Phosphopeptide enrichment was done by titanium dioxide (TiO2) and fractionated by basic reverse phase liquid chromatography (bRPLC) prior to LC-MS/MS analysis on two different mass spectrometrs, Orbitrap Velos and Orbitarp Fusion Tribrid mass spectrometer (ThermoScientific). Peptides identified in triplicates were further used for in-silico functional analysis and validation on large cohort (n=200) by immunohistochemistry on tissue microarray (TMA).
