# Supplementary material for: Phosphorylation of β-catenin at Serine552 correlates with invasion and recurrence of non-functioning pituitary neuroendocrine tumours
Source: Acta Neuropathol Commun. 2022 Sep 16;10:138. doi: 10.1186/s40478-022-01441-5 (PMC9482208; doi:10.1186/s40478-022-01441-5)
Supplement: Supplementary file 4 — Additional File 4: Fig. S3. Differential phosphorylation of Ser, Thr, and Tyr in recurrent NF-PitNETs and reproducibility of data. a–c Fisher’s test shows significantly high number of hyper and hypo phosphorylated Ser (a, p< 0.0001) and Thr (b, p = 0.01) phosphopeptides in R as compared to I. c No difference in number of phosphotyrosine was found. d, e Pearson’s correlation coefficient for invasive (d, r = 0.78) and recurrent (e, r = 0.73) indicate strong reproducibility among replicates (p < 0.0001). I Invasive; R Recurrent; Ser Serine; Thr Threonine; Tyr Tyrosine. [file 40478_2022_1441_MOESM4_ESM.pdf]

Supplementary Fig. 3

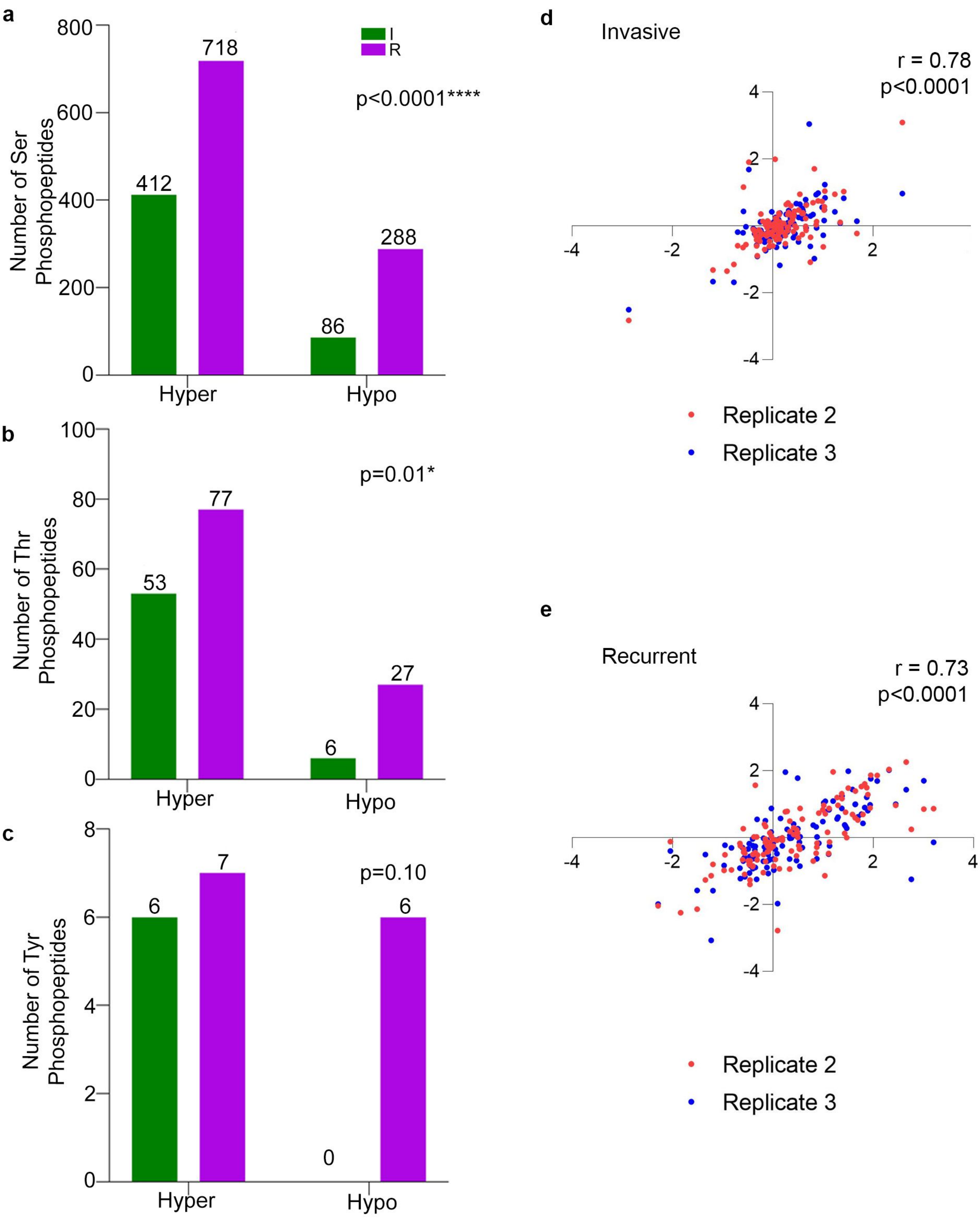

Supplementary Fig. 3 Differential phosphorylation of Ser, Thr, and Tyr in recurrent NF-PitNETs and reproducibility of data. (a-c) Fisher's test shows significantly high number of hyper and hypo phosphorylated Ser (a, $p<0.0001$) and Thr (b, $p=0.01$) phosphopeptides in R as compared to I. (c) No difference in number of phosphotyrosine was found. (d-e) Pearson's correlation coefficient for invasive (d, $r=0.78$) and recurrent (e, $r=0.73$) indicate strong reproducibility among replicates ($p<0.0001$). Abbreviations: I, invasive; R, recurrent; Ser, serine; Thr, threonine; Tyr, tyrosine.
