# Supplementary material for: Phosphorylation of β-catenin at Serine552 correlates with invasion and recurrence of non-functioning pituitary neuroendocrine tumours
Source: Acta Neuropathol Commun. 2022 Sep 16;10:138. doi: 10.1186/s40478-022-01441-5 (PMC9482208; doi:10.1186/s40478-022-01441-5)
Supplement: Supplementary file 5 — Additional File 5: Fig. S4. Increased upregulated phosphopeptides in the recurrent NF-PitNET subgroup. a, b Graphical representation showing the number of phosphopeptides with the Log2-fold change in the various NF-PitNET groups with red lines indicating hypo- and hyper-phosphorylated peptides. c Principle component (PC) analysis of NF-PitNET phosphoproteome reveal disease subgroup segregation and replicates group together. Singular value decomposition (SVD) with imputation of fold change of phosphopeptides of each NF-PitNET subgroup is used to calculate principal components. X and Y axis show principal component 1(PC1) and principal component 2 (PC2) that explain 63.3% and 82.8% of the total variance, respectively. Each grey dots represents an NF-PitNET subgroup. Abbreviations: NI/NR, non-invasive/non-recurrent; I, Invasive; R, Recurrent; PC Principle component. Green colour represents invasive (I) subgroups while blue colour represents recurrent (R). [file 40478_2022_1441_MOESM5_ESM.pdf]

Supplementary Fig. 4

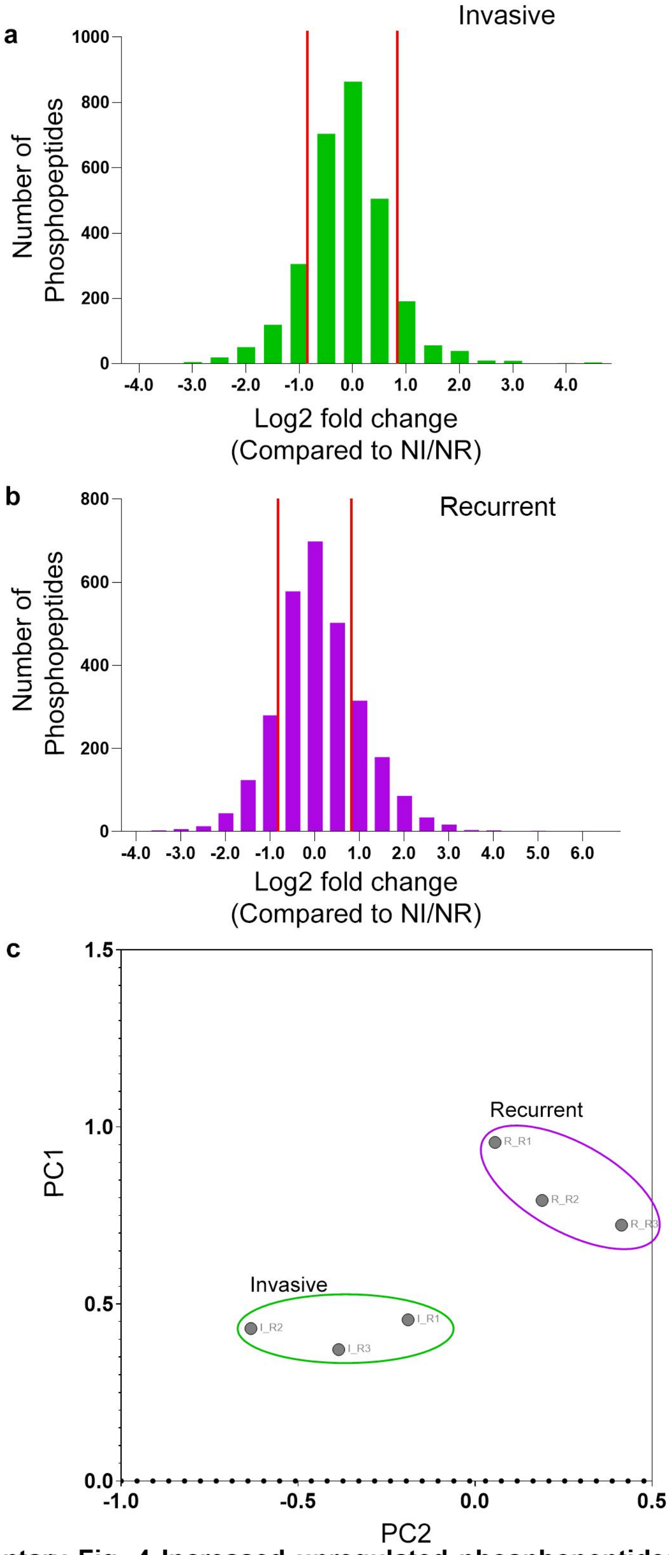

Supplementary Fig. 4 Increased upregulated phosphopeptides in the recurrent NF-PitNET subgroup. (a-b) Graphical representation showing the number of phosphopeptides with the Log_2-fold change in the various NF-PitNET groups with red lines indicating hypo- and hyper-phosphorylated peptides. (c) Principle component (PC) analysis of NF-PitNET phosphoproteome reveal disease subgroup segregation and replicates group together. Singular value decomposition (SVD) with imputation of fold change of phosphopeptides of each NF-PitNET subgroup is used to calculate principal components. X and Y axis show principal component 1(PC1) and principal component 2 (PC2) that explain 63.3% and 82.8% of the total variance, respectively. Each grey dots represents an NF-PitNET subgroup. Abbreviations: NI/NR, non-invasive/non-recurrent; I, invasive; R, recurrent; PC, principle component. Green colour represents invasive (I) subgroups while blue colour represents recurrent (R).
