# Supplementary material for: Phosphorylation of β-catenin at Serine552 correlates with invasion and recurrence of non-functioning pituitary neuroendocrine tumours
Source: Acta Neuropathol Commun. 2022 Sep 16;10:138. doi: 10.1186/s40478-022-01441-5 (PMC9482208; doi:10.1186/s40478-022-01441-5)
Supplement: Supplementary file 6 — Additional File 6: Fig. S5. Gene ontology (GO) pathway analyses showing enriched (red) or depleted (green) hyperphosphorylated proteins in R NF-PitNET. a–d Phosphoproteins exclusively overphosphorylated in recurrent PitNETs were used for gene ontology analysis using FunRich (version 3.1.3) software. X axis represents fold change enrichment of GO categories in recurrent NF-PitNETs as compared to non-invasive/non-recurrent and Y axis represents the GO categories. a Cell component analysis showed endoplasmic reticular membrane proteins were most enriched followed by cell cortex, ribosome, nuclear speck, and cytoplasmic microtubules. b Graphical representation of molecular function revealed proteins with ATPase activity were most enriched while caspase activator proteins were most depleted in recurrent NF-PitNETs. c Graphical representation of biological process showed proteins involved in cell proliferation, regulation of cell proliferation and migration were most enriched. d Graphical representation of biological pathways showed glypican-3 signalling and overall high enrichment of Wnt signalling and regulation of nuclear β-catenin signalling as the most enriched pathways in recurrent NF-PitNETs. R Recurrence; GO Gene ontology [file 40478_2022_1441_MOESM6_ESM.pdf]

Supplementary Fig. 5

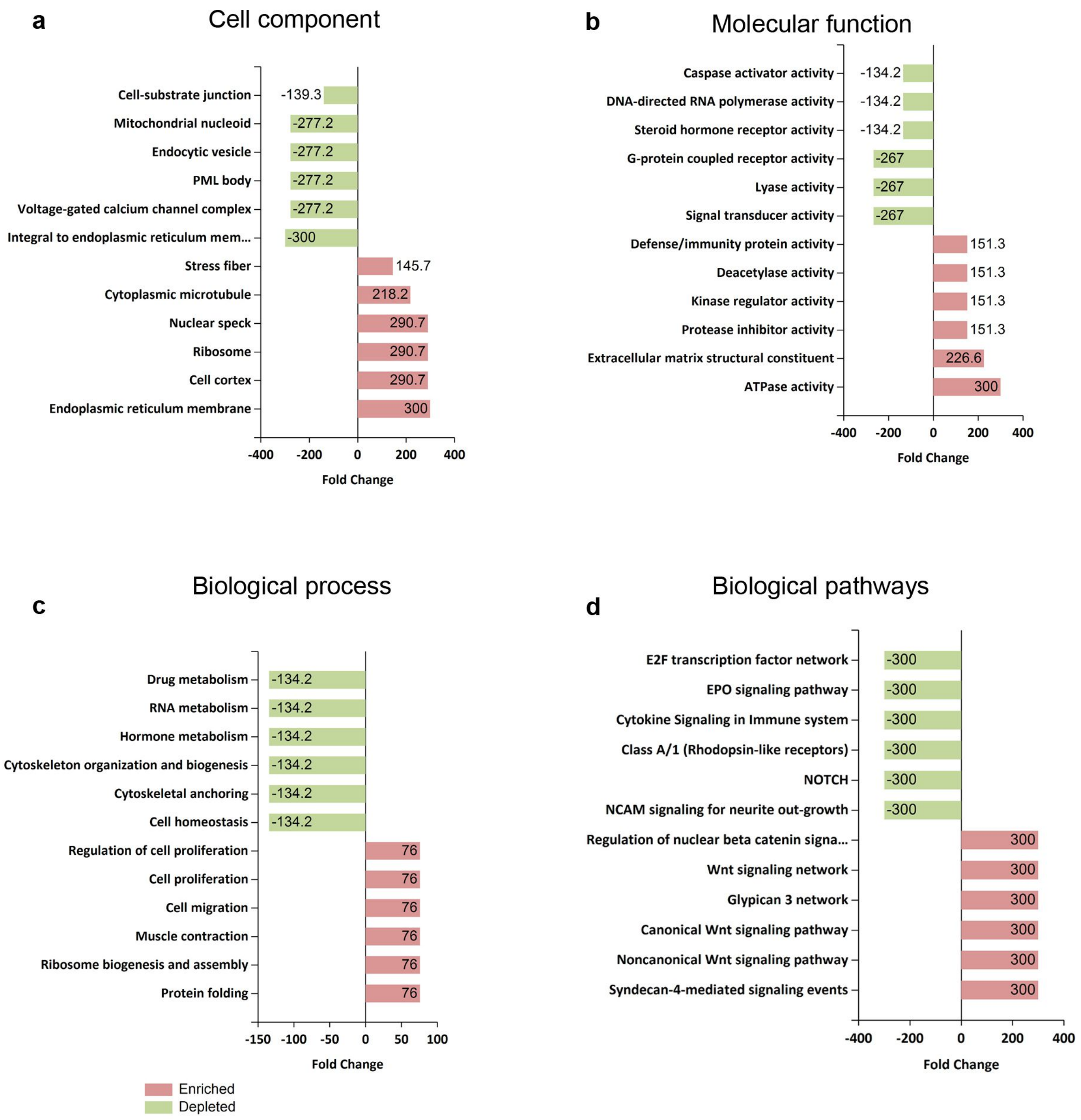

Supplementary Fig. 5 Gene ontology (GO) pathway analyses showing enriched (red) or depleted (green) hyperphosphorylated proteins in R NF-PitNET. (a-d) Phosphoproteins exclusively overphosphorylated in recurrent PitNETs were used for gene ontology analysis using FunRich (version 3.1.3) software. X axis represents fold change enrichment of GO categories in recurrent NF-PitNETs as compared to non-invasive/non-recurrent and Y axis represents the GO categories. (a) Cell component analysis showed endoplasmic reticular membrane proteins were most enriched followed by cell cortex, ribosome, nuclear speck, and cytoplasmic microtubules. (b) Graphical representation of molecular function revealed proteins with ATPase activity were most enriched while caspase activator proteins were most depleted in recurrent NF-PitNETs. (c) Graphical representation of biological process showed proteins involved in cell proliferation, regulation of cell proliferation and migration were most enriched. (d) Graphical representation of biological pathways showed glypican-3 signalling and overall high enrichment of Wnt signalling and regulation of nuclear β-catenin signalling as the most enriched pathways in recurrent NF-PitNETs. Abbreviations: R, recurrence; GO, Gene ontology.
