# Supplementary material for: Phosphorylation of β-catenin at Serine552 correlates with invasion and recurrence of non-functioning pituitary neuroendocrine tumours
Source: Acta Neuropathol Commun. 2022 Sep 16;10:138. doi: 10.1186/s40478-022-01441-5 (PMC9482208; doi:10.1186/s40478-022-01441-5)
Supplement: Supplementary file 7 — Additional File 7: Fig. S6. Protein-protein interaction network of phosphoproteins exclusively overphosphorylated in recurrent NFPTs showed upregulation of β-catenin signalling. Phosphoproteins only overphosphorylated in recurrent subgroup (R) were mapped using FunRich (version 3.1.3) software. Each red node represents a hyperphosphorylated protein and blue line indicates interactions. EGFR as found to be in the center of interaction hub while β-catenin was found to be part of most of the upregulated pathways. Blue arrows indicate β-catenin and MYH9, which are significantly hyperphosphorylated in R. Nodes with green circles represents proteins involved in regulation of nuclear β-catenin signaling, while nodes with yellow circles represents proteins involved in developmental pathways. PSMD2 and PSMA3 (indicated by black arrows) represents proteins involved in cell cycle regulation. [file 40478_2022_1441_MOESM7_ESM.pdf]

Supplementary Fig. 6

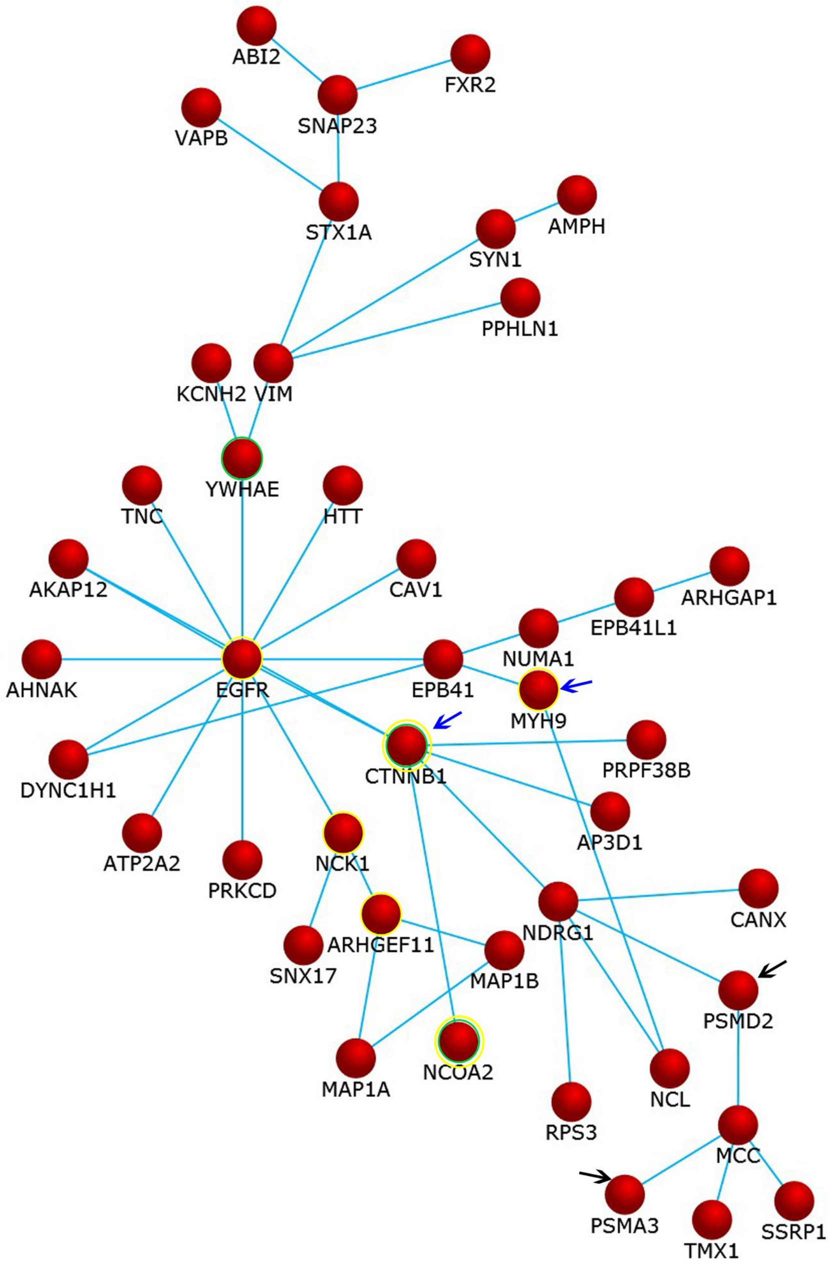

Supplementary Fig. 6 Protein-protein interaction network of phosphoproteins exclusively overphosphorylated in recurrent NFPTs showed upregulation of β-catenin signalling. Phosphoproteins only overphosphorylated in recurrent subgroup (R) were mapped using FunRich (version 3.1.3) software. Each red node represents a hyperphosphorylated protein and blue line indicates interactions. EGFR as found to be in the center of interaction hub while β-catenin was found to be part of most of the upregulated pathways. Blue arrows indicate β-catenin and MYH9, which are significantly hyperphosphorylated in R. Nodes with green circles represents proteins involved in regulation of nuclear β-catenin signaling, while nodes with yellow circles represents proteins involved in developmental pathways. PSMD2 and PSMA3 (indicated by black arrows) represents proteins involved in cell cycle regulation.
